# False discovery rate control for grouped hypotheses: application to miRNAome data

**DOI:** 10.7717/peerj.21257

**Published:** 2026-05-25

**Authors:** Nilanjana Laha, Salil Koner, Austin Labowitz, Navonil De Sarkar

**Affiliations:** 1 Department of Statistics, Texas A&M University, College Station, United States of America; 2 Department of Statistics, University of California, Riverside, United States of America; 3 Department of Pathology, Medical College of Wisconsin, Milwaukee, WI, United States of America

**Keywords:** Oral squamous cell carcinoma, False discovery rate, MiRNA deregulation, Benjamini Hochberg method, Grouped Benjamini Hochberg, Multiple hypothesis testing

## Abstract

Bioinformatics studies often involve numerous simultaneous statistical tests, increasing the risk of false discoveries. To control the false discovery rate (FDR), these studies typically apply a statistical method called the Benjamini–Hochberg (BH) method. However, BH can be overly conservative, particularly in small-sample studies, and it does not take advantage of relevant structural information among the hypotheses, such as groupings. Group structures can arise, for example, when genomic features located in close proximity are co-regulated. Recent statistical developments have yielded group-adaptive BH methods that can leverage pre-existing group information to improve statistical power while maintaining FDR control. However, these methods remain underutilized in bioinformatics practice. In this study, we illustrate the practical application of group-adaptive BH methods using a previously published, moderately scaled microRNA (miRNA) dataset. Even under simple groupings based on chromosomal location, these methods identified more miRNAs with significantly deregulated expression (FDR-adjusted *p*-value < 0.05) compared to the traditional BH method. Most of the new discoveries are supported by prior literature and a related 2017 study. Although sensitivity to grouping strategy varied across methods, our control analysis indicated that, for most methods, the additional detections may be attributable to the incorporation of group information. Our results highlight the potential of specialized BH methods for controlling the FDR in omics studies with pre-defined group structures, and motivate further evaluation of their generalizability across diverse datasets.

## Introduction

Molecular profiling technologies, including sequencing, microarrays, and targeted panel assays, enable the simultaneous measurement of many molecular features in biological samples (Dudoit, Shaffer & Boldrick, 2003; Goeman & Solari, 2014; Sesia et al., 2021). Analyzing such data typically involves testing many hypotheses in parallel, each corresponding to one biological element, such as one gene expression in a microarray or RNA-seq study. The rejection of a hypothesis is generally associated with biologically relevant elements, indicating statistical “discovery.” However, testing many hypotheses simultaneously inflates the probability of false positives, making it necessary to apply statistical procedures that control false discoveries (Menyhart, Weltz & Gyrffy, 2021). A widely used approach for this purpose is controlling the false discovery rate (FDR) (Korthauer et al., 2019). The FDR is the expected proportion of false rejections among all rejected hypotheses. An FDR of 5% implies that, on average, five of 100 significant findings are expected to be false positives.^1^ 1A preprint version of this manuscript was previously posted on bioRxiv (Laha et al., 2025).

In the past decades, various statistical methods have been proposed for controlling the FDR. However, the Benjamini and Hochberg (BH) procedure remains the most prominent tool for this purpose in the realms of bioinformatics (Benjamini & Hochberg, 1995; Korthauer et al., 2019). Originally developed for independent hypotheses, the BH method also controls the FDR under weak dependence, an assumption that often holds in biological applications (Benjamini & Yekutieli, 2001). The BH method treats all hypotheses as exchangeable and applies a common *p*-value threshold, rejecting only those hypotheses with *p*-values below this cutoff. Although the BH method reliably controls the FDR, the use of a common *p*-value threshold forces it to be uniformly stringent across all hypotheses (Ignatiadis & Huber, 2021; Korthauer et al., 2019). This issue is exacerbated when the number of hypotheses greatly exceeds the sample size, which is a common issue in high-dimensional biological studies (Hu, Zhao & Zhou, 2010; Li & Barber, 2019). In such settings, the large number of simultaneous tests increases the number of spurious findings, forcing BH to use more stringent rejection thresholds to maintain FDR control. This increased stringency can make weaker signals harder to detect (Bourgon, Gentleman & Huber, 2010; Ignatiadis et al., 2016). As a result, the conservativeness of the BH method can hinder scientific discovery in these regimes.

The BH method treats all hypotheses as exchangeable because it does not incorporate auxiliary information about the hypotheses (Genovese, Roeder & Wasserman, 2006). In many domains, including bioinformatics, such information can be available. This study focuses on hypotheses with pre-existing group structures, which often arise naturally in biological data. For example, closely located genes are frequently correlated and may be co-expressed due to shared transcription programs and regulatory mechanisms, forming positional clusters (Koutná et al., 2007).

Recent advancements in statistics have led to modified BH methods that can leverage pre-existing group structures, *e.g.*, the grouped BH (GBH) methods of Hu, Zhao & Zhou (2010) and the structure adaptive BH algorithm (SABHA) of Li & Barber (2019).^2^ 2SABHA can adapt to a wide range of structures, including, but not limited to, grouped hypotheses (Li & Barber, 2019).Such methods recalibrate the *p*-values, prioritizing rejections from potential critical groups. A group’s criticality is determined by the proportion of true null hypotheses within it; smaller proportions indicating greater criticality. When pre-existing group structure is present, these *group-adaptive BH* methods were shown to achieve higher statistical power than the BH method in prior controlled experiments (Korthauer et al., 2019; Hu, Zhao & Zhou, 2010; Li & Barber, 2019). A higher statistical power indicates the ability to detect lower signals, thus potentially increasing the number of scientific discoveries. Nevertheless, these group-adaptive BH methods are yet to see widespread adoption in bioinformatics practice.

There exist many other FDR control methods that adapt to additional information, such as estimates of the proportion of null hypotheses or auxiliary covariates, to improve power. Methods that adapt to the estimated proportion of true null hypotheses include Storey’s *q*-value procedure (Storey, 2002), adaptive step-up procedures (Benjamini, Krieger & Yekutieli, 2006; Blanchard & Roquain, 2008), and the two-stage BH procedure (Benjamini & Hochberg, 2000), among others. Methods that leverage side information in the form of informative covariates include independent hypothesis weighting (IHW, Ignatiadis et al., 2016), independent filtering (Bourgon, Gentleman & Huber, 2010), false discovery rate regression (FDRreg, Zhang, Xia & Zou, 2019), and adaptive *p*-value thresholding (AdaPT, Lei & Fithian, 2018), to name a few. Methods that adapt to effect size and variance estimates include, but are not limited to, the adaptive shrinkage method (Ash, Stephens, 2017) and the heteroskedasticity-adjusted multiple testing method (HAMT, Gang & Banerjee, 2025). An emerging statistical framework based on knockoff constructions has also shown promise for FDR control and is flexible enough to incorporate group structures (Sesia et al., 2021). A comprehensive review of existing FDR methods can be found in Korthauer et al. (2019).

However, most of these methods are typically designed to handle broad classes of dependence structures and are not specific to pre-existing group structures (Li & Barber, 2019; Friston, 2003). Moreover, methods such as IHW, independent filtering, FDRreg, and AdaPT, require informative covariates that are independent of the *p*-values under the null hypothesis (Ignatiadis et al., 2016; Korthauer et al., 2019). These covariates need to be chosen carefully, often using domain knowledge, as violation of the independence requirement can lead to loss of FDR control (Ignatiadis et al., 2016; Korthauer et al., 2019). Since our primary aim is to illustrate how grouping improves power while controlling the FDR, we restrict our attention to group-adaptive BH methods, especially GBH and SABHA, to keep the scope of the study coherent and allow for a clearer interpretation of the results. Notably, these methods do not use groups as the unit of rejection. Instead, rejections are conducted at the level of the individual hypotheses. However, FDR control methods that reject hypotheses at the group level are also available (Efron & Tibshirani, 2002; Benjamini & Heller, 2007).

This work is an applied study illustrating the integration of group-adaptive BH procedures into moderate-throughput microarray analyses with pre-specified group structures. To this end, we use the microRNA (miRNA) dataset from De Sarkar et al. (2014), in which one of the present co-authors was involved. The dataset comprises 522 miRNAs measured using a targeted quantitative polymerase chain reaction (qPCR) panel from 18 cancer patients with gingival buccal squamous cell carcinoma (GBSCC), a subtype of oral squamous cell carcinoma (OSCC). Although modest in scale compared to modern RNA-seq experiments, this dataset is still high-dimensional, with the number of assays far exceeding the sample size, and is representative of settings where targeted panels are applied to limited cohorts. Addressing the challenge of small sample size (*n* = 18) required careful analysis choices, and the pipeline presented here may serve as a useful template for similar studies. Such contexts remain common in translational and clinical studies (Bustin et al., 2009). Because the discussed procedures require only pre-specified group structures, the encouraging results from this dataset suggest that they may also be useful in larger-scale, high-dimensional bioinformatics studies where similar group information is available.

De Sarkar et al. (2014)’s goal was to investigate the role of miRNAs in GBSCC by comparing their expression in histopathologically confirmed malignant and healthy normal (control) tissues. As there were 522 miRNA assays, 522 t-tests were performed. De Sarkar et al. (2014) used the BH method to control the FDR at 5% level of significance. It identified seven miRNAs to be significantly deregulated in the malignant tissues. However, De Sarkar et al. (2014) noticed that the expression levels of some cancer biomarkers, including hsa-miR-21-5p and hsa-miR-1, were either borderline or below the BH threshold. Some target genes of these miRNAs were found to be significantly deregulated in the opposite direction in a follow-up whole transcriptome analysis study by Singh et al. (2017). This study analyzed a case series substantially overlapping with that of De Sarkar et al. (2014). This observation raised the question of whether the BH method had sufficient statistical power to detect deregulated miRNAs in their dataset. De Sarkar et al. (2014)’s dataset therefore serves as a suitable case study for evaluating whether group-adaptive FDR methods can improve the detection of biologically relevant miRNAs.

Considering our limited sample size (*n* = 18), the number of miRNAs analyzed (*p* = 522), and their distribution across the genome, we opted for a relatively straightforward grouping approach in this proof-of-concept analysis. Because proximal miRNAs have been reported to exhibit co-expression (Koutná et al., 2007), the physical location of the miRNAs on the chromosome was used as the basis for grouping. While overly simplistic, this strategy was suitable for the small sample size and serves our purpose of illustrating how incorporating external biological information can improve detection. Even under this simple criterion, multiple grouping schemes are possible. We compared several such schemes, which were defined solely by genomic position and did not rely on expression data. Then we demonstrated the application of group-adaptive BH methods under these schemes and compared their performance with the BH method. A small control study was also conducted using randomly assigned groups. We used existing literature and the whole transcriptome analysis of Singh et al. (2017) to discuss the relevance of the additional miRNAs discovered by the group-adaptive BH methods.

## Material and Methods

‘Study participants and miRNAome dataset’ provides a brief overview of the study population and the resulting miRNAome dataset. ‘Pairwise t-tests and Group-adaptive BH methods’ describe the implementation of the *t*-tests and the group-adaptive BH methods, respectively. ‘Grouping schemes’ discusses various position-based schemes for grouping the miRNAs.

### Study participants and miRNAome dataset

We used publicly available data from De Sarkar et al. (2014). The study participants were 18 unrelated Indians aged 39–80 years with tobacco habits, with a male:female ratio of 5:4. All patients had histopathologically confirmed GBSCC, which is a type of OSCC prevalent in the tobacco-chewing population in South Asia. The demographic characteristics of the study population are summarized in Table S3.2 in the Supplement. The dataset contained ΔCt values for 522 miRNAs derived from 18 tumor-normal sample pairs of the 18 patients. ΔCt values serve as surrogate estimates for relative miRNA expression levels (relative to geometric mean expression of three endogenous control genes) tissues. The ΔCt values were used to obtain the ΔΔCt values, where ΔΔCt = ΔCt of a miRNA in cancer tissue −ΔCt of that miRNA in control tissue. Further details on the data collection methods are provided in Section S1 of the Supplement.

#### Missingness

Some miRNAs were not expressed in all 18 patients in De Sarkar et al. (2014)’s dataset. In their study, miRNA expression profiling was performed using TaqMan Low-Density Arrays (TLDA-A V2 and TLDA-B V3) on the 7900HT FAST Real-Time PCR System (Applied Biosystems, USA), equipped with a TLDA flat block. In qPCR, when an amplification reaction fails to reach the fluorescence detection threshold within a pre-specified number of PCR cycles, the corresponding miRNA measurements are considered non-informative technical noise, *i.e.,* a missing value (McCall et al., 2014). Such failure is common for many miRNAs, especially circulating miRNAs, since they often exist in plasma at low concentrations (De Ronde et al., 2017). Therefore, a high proportion of missing observations is expected for qPCR-based miRNA profiling studies (De Ronde et al., 2017; Gevaert et al., 2018; McCall et al., 2014).

 De Sarkar et al. (2014) set the maximum number of PCR cycles (Ct cutoff) to 40, which is a typical choice in qPCR-based miRNA assays (McCall et al., 2014). If either the tumor or the matched normal expression value for a given miRNA surpassed the Ct cutoff, De Sarkar et al. (2014) excluded both values to simplify downstream analyses. Consequently, tumor-control pairs for several miRNAs were missing in the miRNA dataset. While the detection limit inherent to qPCR is likely a contributor to the missingness, other technical or biological factors may also have played a role. Nonetheless, the missingness in the miRNAome measurements appears to be not at random (MNAR) (Hughes et al., 2019; McCall et al., 2014).

In what follows, a missing pair indicates that the miRNA expression was missing for both the normal and the corresponding tumor tissue for a given patient. Each patient had at least 12 missing miRNA expression pairs, although most had fewer than 50 missing cancer-normal pairs. Figure S2.1 displays the number of missing miRNA pairs for each patient, and Figure S2.2 shows the histogram of the number of missing pairs across patients. Overall, 59.77% of miRNAs had at least one missing pair, 30% had more than two missing pairs, and approximately 20% of miRNAs had missingness in more than 20% of samples. None had more than 50% missing pairs, as miRNAs exceeding this threshold were removed during data pre-processing by De Sarkar et al. (2014). Comparable levels of missingness have been reported in other qPCR-based miRNA studies (Gevaert et al., 2018; Mooney et al., 2015; Ravaggi et al., 2024). For further details on missingness, refer to Section S2.

De Sarkar et al. (2014) imputed the missing observations using an imputation strategy based on sample medians in their statistical analysis. However, we observed a sharp increase in the number of discoveries regardless of the FDR control method when using simplistic imputation strategies such as median or mean imputation. This is unsurprising because it is well-known that imputation with the median or mean can artificially decrease the sample variance, especially when the sample size is small, leading to smaller *p*-values (Zhang, 2016; Sarkar & Zhao, 2022). This can increase the risk of false discoveries for test units both with or without missing values. Both the BH procedure and its variants rely on ranking *p*-values to determine an adjusted *p*-value threshold. When imputation results in smaller *p*-values, the adjustment mechanism can yield a larger overall threshold (cf., *e.g.*, Benjamini & Hochberg, 1995 for details on the adjustment). For example, in our dataset, the BH-corrected *p*-value threshold increased from 0.00020 in the non-imputed data to 0.00094 under median imputation. This means that, in the non-imputed data, BH considered miRNAs with *p*-value ≤0.00020 as significantly deregulated, whereas after imputation, BH considered any miRNA with *p*-value ≤0.00094 to be significantly deregulated. Since the group-adaptive BH methods are variants of BH, they also became more lenient under median imputation.

A more sophisticated imputation strategy may reduce the risk of imputation-driven false detections. However, given our limited sample size, the relatively high proportion of missing values, and the complexity of measuring all biological factors behind miRNA expression, we chose not to impute missing values. Instead, we conducted our statistical analysis using only complete cases. Finally, since our groups are informed by external biological information, they are unaffected by data missingness.

### Pairwise t-tests

The t-tests were conducted as in De Sarkar et al. (2014); details are provided here for completeness. Upregulation or downregulation of miRNA expression was determined by checking if the change of miRNA expression exceeded 2 fold compared to the control. The fold-changes are measured by ΔΔCt value: a positive ΔΔCt implies >2 fold downregulation, while a negative ΔΔCt implies >2 fold upregulation of miRNA expression. We performed 522 one-sided pairwise t-tests to determine whether the expression of miRNAs was significantly deregulated. For each miRNA, the direction of the one-sided paired *t*-test was determined by the sign of the median ΔΔCt value across the 18 patients. The null hypothesis of the one-tailed paired *t*-test was that the expression of a particular miRNA is not greater than 2 fold upregulated (or downregulated).

### Group-adaptive BH methods

As noted in ‘Introduction’, group-adaptive BH methods assign greater importance to groups with a higher estimated proportion of true null hypotheses. The group-wise null proportions can be estimated using various statistical approaches. Examples include the two-stage step-up (TST) method (Benjamini, Krieger & Yekutieli, 2006), the least slope limited (LSL) method (Benjamini & Hochberg, 2000), and likelihood-based approach (Li & Barber, 2019), which lead to TST-GBH, LSL-GBH, and the group-aware version of SABHA, respectively (Hu, Zhao & Zhou, 2010; Li & Barber, 2019). As mentioned in ‘Introduction’, we focus on these three group-adaptive BH methods and compare them with the standard BH procedure. When the *p*-values are independent or weakly correlated, these methods were shown to control the FDR if group-wise null proportions are reasonably well-estimated, Hu, Zhao & Zhou (2010) and Li & Barber (2019). If the groups are similar, *i.e.,* if the proportion of true null hypotheses does not vary across groups, then SABHA, TST-GBH, and LSL-GBH reduce to Storey’s adaptive BH (Storey, 2002), the TST BH (Benjamini, Krieger & Yekutieli, 2006), and the LSL BH (Benjamini & Hochberg, 2000), respectively. These procedures are adaptive variants of BH that estimate the proportion of null hypotheses and use this estimate to modify the BH rejection threshold. TST-GBH and LSL-GBH have previously been used in applied contexts (cf. Sankaran & Holmes, 2014). Especially, Zhang et al. (2013) applied them to identify differentially methylated regions in the human genome, and Liu et al. (2015) used them to detect differentially expressed unigenes during the blooming process of Asteraceae flowers.

**Implementation:** TST-GBH and LSL-GBH were implemented following the algorithms provided by Hu, Zhao & Zhou (2010). To implement SABHA, we used the R codes provided by the authors of the corresponding paper by Li & Barber (2019). SABHA requires two tuning parameters: (1) *ϵ*, a lower bound on the proportion of the true null hypotheses, and (2) a threshold *τ*, used for calibrating the individual *p*-value thresholds. Similar to Li & Barber (2019), we used *ϵ* = 0.1 and *τ* = 0.5. We found that the discoveries were not sensitive to small perturbations of either of these tuning parameters. We implemented BH using the package Stats in the software R. Our code is provided at the GitHub repository https://github.com/nilanjanalaha/GABH.git.

### Grouping schemes

As noted earlier, miRNAs were grouped by chromosomal location. While co-expression may arise from other biological factors, this simple scheme was appropriate for the small sample size (*n* = 18) relative to the number of genes (∼500) in this proof-of-concept analysis. The miRNAs can also be grouped using data-dependent clustering methods such as k-means clustering, tight clustering (Karmakar et al., 2019), and weighted gene co-expression network analysis (WGCNA) (Horvath, 2011). However, recent studies highlighted the risk of potentially elevated type-I error when using the same data for clustering and downstream testing (Gao, Bien & Witten, 2020; Luecken & Theis, 2019; Lähnemann et al., 2020). Given our limited sample size, reusing the miRNA expression data for group assignment would further exacerbate this risk.

Even when grouping miRNAs by spatial location on the chromosome, multiple grouping schemes/ strategies are possible. We considered hierarchical schemes, starting with the coarsest scheme, in which miRNAs were grouped solely by chromosome number, and progressively splitting the groups to obtain finer schemes. The first three schemes were: (a) grouping by chromosome number (miRNAs on the same chromosome form one group); (b) grouping by chromosome number and strand (miRNAs on the same chromosome and strand form one group); and (c) grouping by chromosome number, strand, and arm (miRNAs on the same chromosome, strand, and chromosomal arm form one group). Groups with no miRNAs among the 522 assayed were excluded, and single-miRNA groups were merged with the nearest neighboring group.

To obtain finer groupings from Scheme (c), we generated multiple new grouping schemes by splitting large groups in Scheme (c) according to different maximum group sizes, where group size refers to the number of miRNAs in a group. Specifically, for each *k* ∈ {25, 23, …, 5}, we formed a new scheme in which any Scheme (c) group with more than *k* members was divided into adjacent subgroups of size *k*. The chromosome coordinates provided by De Sarkar et al. (2014) were used to guide splitting, ensuring that adjacent miRNAs were grouped together. If a group size was not a multiple of *k*, the final subgroup was allowed to have fewer than *k* members. For simplicity, we may refer to this family of refinements as “grouping schemes derived from Scheme(c)”.

**Figure 1:**
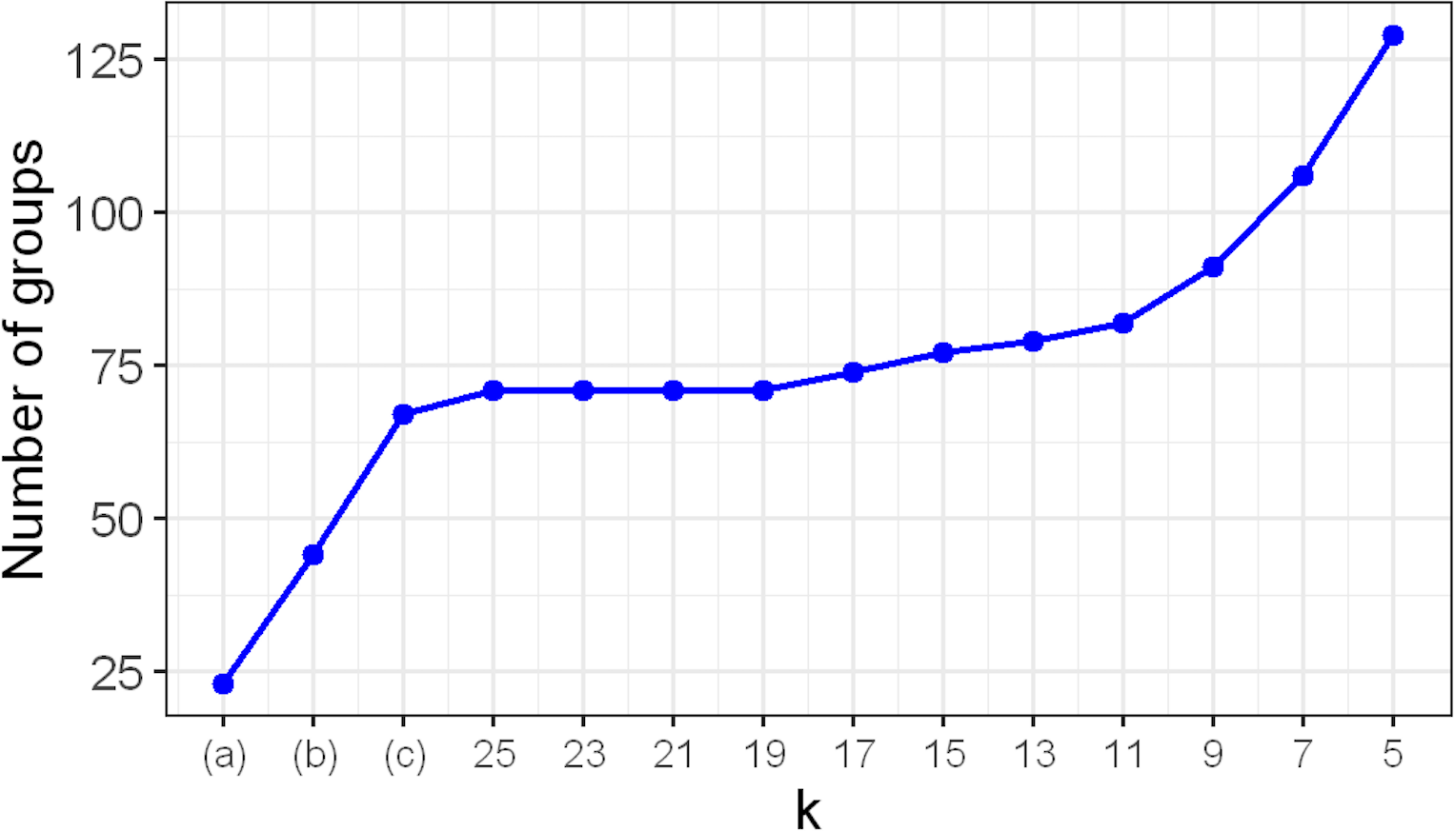
Plot of the total number of groups as a function of grouping schemes. The grouping schemes (A), (B), and (C) are described in ‘Grouping schemes’. The schemes corresponding to *k* = 5, …, 25 are finer partitions obtained by further splitting the groups in Scheme (C), as detailed in ‘Grouping schemes’. The *y*-axis represents the total number of groups under each scheme.

As the grouping schemes become progressively finer from Scheme (a) to *k* = 5, the total number of groups per scheme increases accordingly. Figure 1 plots the total number of groups for each scheme. Schemes (a), (b), and (c) have 23, 44, and 67 groups, respectively, rising to 129 at *k* = 5. We did not consider schemes with *k* ≤ 4 because the number of groups becomes excessively large. For instance, *k* = 2 yields 278 groups. This is problematic because, as we shall see in ‘Results’, the GBH methods tend to become too liberal when the number of groups is large. The minimum group size was three for Scheme (a) and two for all other schemes.

Table S3.1 in the Supplement tabulates the sizes and locations of the groups in Scheme (c). Figure S3.1 in the Supplement displays the histograms of the group sizes for Schemes (a), (b), (c), and when *k* = 15 and 5. Figure 1 indicates that the number of groups changes little when Scheme (c) is split into finer groups up to *k* = 11, but increases sharply when *k* falls below 11. This occurs because Scheme (c) contains only a few large groups. Eleven groups, which is about only 16% of all groups in Scheme (c), have size ≥ 11. In contrast, this scheme contains a larger proportion of moderately sized groups, with 17 groups (about 25%) with size ≥ 9 (see also Table S3.1 and Figure S3.1ii).

#### Intra- and inter-group association

Hughes et al. (2019) observed that group-adaptive BH methods exhibit higher statistical power than the standard BH method when the intra-group association, that is, the association among test units within the same group, is stronger than the inter-group association, which refers to the association between test units in different groups. Visual inspection of pairwise correlations between miRNAs’ ΔΔCt values suggests that correlations within groups are generally higher or comparable to those across groups. Figure S3.2 in the Supplement illustrates this pattern for several groups under Scheme (c). This figure also shows that the contrast between intra- and inter-group correlations is more pronounced for the position-based groups than for pseudo-groups formed by randomly selecting miRNAs.

For each of our grouping schemes, we heuristically compared the intra-group and inter-group correlations between the ΔΔCt values utilizing a simple random effect model, which is a widely used statistical approach (Nakagawa, Johnson & Schielzeth, 2017). Details on fitting this model and the corresponding quantification of inter-group and intra-group correlations are provided in the ‘Appendix’. The simple random effect model assumes the inter-group and intra-group correlations to be constant across all groups. Even if this assumption is violated, the estimated correlations provide meaningful assessments of the overall strength of the inter-group and intra-group association. Figure S3.3 in the Supplement shows the estimated correlations across our grouping schemes. The estimated inter-group correlation ranged between 0.041 and 0.044 for all grouping schemes, indicating a near-constant and weak inter-group association. In contrast, the intra-group correlation estimates were 0.108, 0.119, and 0.139 for schemes (a), (b), and (c), respectively, and ranged between 0.147 and 0.18 for all other grouping schemes. Thus, the simple random effect model suggested a weaker inter-group association compared to intra-group association for our grouping schemes. Figure S3.3 in the Supplement also shows that the intra-group correlation estimates generally increase as the grouping scheme becomes finer. This is unsurprising because intra-group correlation increases when within-group variation decreases. Within-group variation is expected to decrease when the grouping becomes finer (Searle, Casella & McCulloch, 2009).

## Results

‘Significantly deregulated miRNAs’ presents detection patterns of the group-adaptive BH methods under different grouping schemes. ‘Control study: random group assignment’ reports a control study with randomly assigned groups. ‘Relevance of the novel miRNAs’ discusses the biological relevance of the additional miRNAs identified by the group-adaptive BH methods.

### Significantly deregulated miRNAs

**Figure 2:**
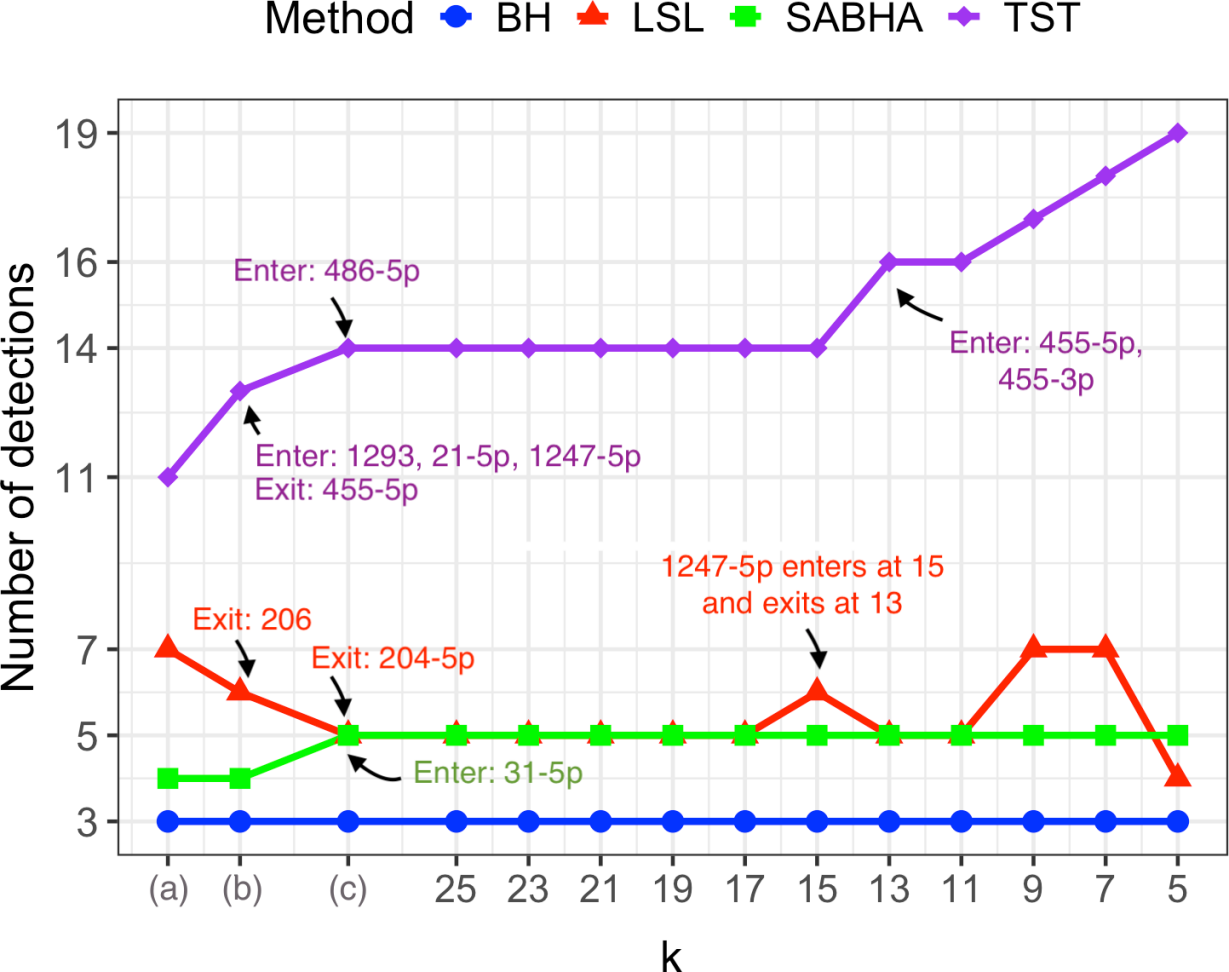
Plot of the number of detections by each FDR control method *vs* grouping scheme. The methods compared were BH, TST-GBH (abbreviated as TST), LSL-GBH (abbreviated as LSL), and SABHA, each applied with an FDR control level of *α* = 5%. The grouping schemes (A), (B), and (C) are as described in ‘Grouping schemes’. The schemes corresponding to *k* = 5, …, 25 are finer partitions obtained by further splitting the groups in Scheme (C), as detailed in ‘Grouping schemes’. The *y*-axis shows the total number of detections from each grouping scheme. Detection sets changed little from one scheme to the next, except for a few miRNAs entering or leaving. For Schemes (A), (B), and (C), as well as the schemes with *k* = 13, …, 25, entries and departures are marked in the plot. When a new miRNA entered a detection set, its name is listed under “Enter”; when an miRNA exited, its name is listed under “Exit.” Since BH is not group-dependent, there are no entries or exits.

In this section, we compare the significantly deregulated miRNAs detected by BH, TST-GBH, LSL-GBH, and SABHA. All methods were applied at a 5% level of significance. BH identified three miRNAs, hsa-miR-133a-3p, miR-31-3p, and miR-206, as significantly deregulated. The detections of the group-adaptive BH methods depended on the grouping scheme but consistently included these three miRNAs, except under Scheme (b), where LSL-GBH did not detect hsa-miR-206. Figure 2 shows the number of significantly deregulated miRNAs for each method across grouping schemes. TST-GBH was the most liberal of the four methods, yielding the highest number of detections for any scheme, consistent with the observations of Sarkar & Zhao (2022). The number of detections by TST-GBH and SABHA generally increased as the grouping scheme became finer, whereas LSL-GBH showed no consistent trend.

Among the group-adaptive BH methods, SABHA was the most conservative and most robust to changes in grouping schemes, followed by LSL-GBH. As shown in Fig. 2, SABHA’s detections were the most similar to BH’s among the three group-adaptive methods. Under the two coarsest schemes, *i.e.,* Schemes (a) and (b), SABHA’s detections differed from that of BH only by the inclusion of hsa-miR-1. Under Scheme (c) and the finer schemes derived from it, only hsa-miR-31-5p joined SABHA’s detections. Figure 2 shows that the set of significantly deregulated miRNAs varied minimally for all methods across the derived grouping schemes when the maximum group size *k* was in {11, 13, …, 25}, differing by at most two miRNAs. This stability is unsurprising because the group compositions are nearly identical for *k* ∈ {11, 13, …, 25} (see ‘Grouping schemes’). In contrast, when the maximum group size dropped below 10, detections by TST-GBH—and, to a lesser extent, LSL-GBH—became more sensitive to the choice of grouping scheme. In other words, the plot of the number of discoveries *vs* grouping schemes for the GBH methods exhibits *a moderate elbow* at *k* = 10. This probably occurs because, for *k* ≤ 10, both the number of groups and the estimated intra-group correlation rise more sharply with decreasing *k* (Fig. 2; Fig. S3.3).

**Figure 3:**
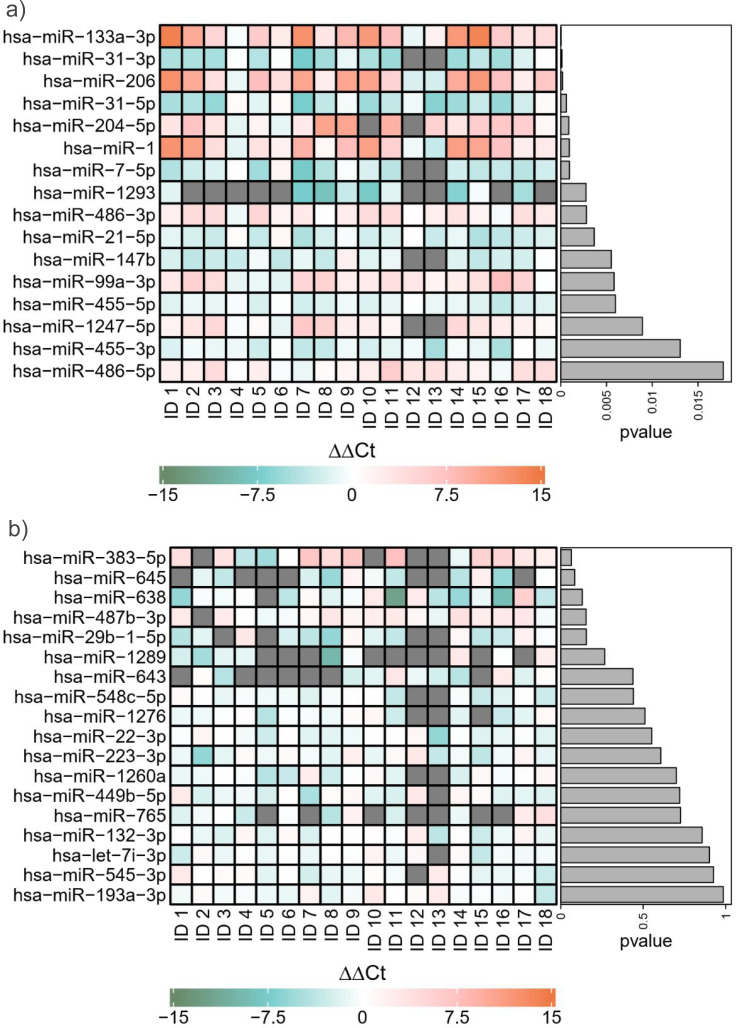
Heatmap of ΔΔ*Ct* values. The heatmap illustrates patterns of miRNA expression deregulation in cancer tissues from 18 GBSCC patients. (A) corresponds to the 16 deregulated miRNAs reported in Table 1, while (B) shows a control group of 16 miRNAs randomly selected from the complement of these deregulated miRNAs. The ΔΔ*Ct* values in (A) exhibit visibly larger magnitudes than those in the control group. The miRNAs are ordered as per unadjusted *p*-values (indicated as “pval”) obtained from the paired t-tests. Missing values are shown in grey.

In view of the elbow at *k* = 10, we restrict our focus to miRNAs detected as significantly deregulated by at least one FDR control method under schemes (a), (b), (c), or any derived scheme with *k* ∈ {11, 13, …, 25}. This yields a total of sixteen miRNAs, listed in Table 1. Among these significantly deregulated miRNAs, hsa-miR-31-3p/5p, miR-7-5p, miR-21-5p, miR-147b, miR-1293, and miR-455-3p/5p were upregulated, while miR-133a-3p, miR-206, miR-204-5p, miR-1, miR-486-3p/5p, miR-99a-3p, and miR-1247-5p were downregulated. The list of miRNAs detected only under Scheme (a) or Scheme (b) is provided in Table S4.1. Figure 3 compares the heatmap of ΔΔ*Ct* values for the sixteen significantly deregulated miRNAs with that of a control group of an equal number of non-deregulated miRNAs. The control miRNAs were randomly selected from the complement of the significantly deregulated set. The significantly deregulated miRNAs exhibit a stronger deregulation pattern than the control group.

**Table 1:** Table of significantly deregulated miRNAs under grouping Scheme (c) and the derivative schemes. The FDR control methods are BH, TST-GBH (TST), LSL-GBH (LSL), and SABHA. The listed miRNAs were detected as significantly deregulated (5% significance level) by at least one method when the grouping scheme was either Scheme (c) or a derivative scheme obtained from it. Here, the derived schemes correspond to *k* = 5, …, 25 (cf. ‘Grouping schemes’). If a method detected an miRNA under all grouping schemes, its row is marked with “x”; otherwise, the row lists the schemes where it was detected. The “Chr.” column gives the chromosome, the “Str.” column indicates the chromosome strand, and the “arm” column specifies the arm. The “ΔΔCt” column provides the average ΔΔCt value for each miRNA across 18 patients. The “*p*-value” column shows raw *p*-values from paired t-tests. Rows are sorted by *p*-values, and the arrows following them denote whether the expression was upregulated (↑) or downregulated (↓) according to the sign of ΔΔCt. The “Missing pairs” column provides the number of missing pairs of observations for each miRNA.

**miRNA**	**Chr.**	**Str.**	**Arm**	ΔΔCt	***p*-value**	Missing pairs	**BH**	**TST**	**LSL**	**SABHA**
hsa-miR-133a-3p^*^	18	–	q	6.7	5.32*E*^−05^↓	0	x	x	x	x
hsa-miR-31-3p^*^	9	–	p	−3.8	1.31*E*^−04^↑	2	x	x	x	x
hsa-miR-206^*^	6	+	p	6.0	1.97*E*^−04^↓	0	x	x		x
hsa-miR-31-5p^*^	9	–	q	−3.4	6.18*E*^−04^↑	0		x	x	x
hsa-miR-204-5p^*^	9	–	q	4.6	8.81*E*^−04^↓	2		x		
hsa-miR-1	18	–	q	5.2	9.38*E*^−04^↓	0		x	x	x
hsa-miR-7-5p^*^	15	+	q	−3.1	9.50*E*^−04^↑	2		x	x	
hsa-miR-1293^*^	12	–	q	−4.8	2.77*E*^−03^↑	9		x		
hsa-miR-486-3p	8	–	p	2.4	2.81*E*^−03^↓	0		x		
hsa-miR-21-5p	17	+	q	−2.2	3.67*E*^−03^↑	0		x		
hsa-miR-147b	15	+	q	−2.1	5.52*E*^−03^↑	2		x		
hsa-miR-99a-3p	21	+	q	2.8	5.83*E*^−03^↓	0		x		
hsa-miR-455-3p	9	+	q	−1.7	5.98*E*^−03^↑	0		*k* = 11, 13		
hsa-miR-1247-5p	14	–	q	2.4	8.93*E*^−03^↓	2		x	*k* = 15	
hsa-miR-455-5p	9	+	q	−1.4	1.30*E*^−02^↑	0		*k* = 11, 13		
hsa-miR-486-5p	8	–	p	2.0	1.78*E*^−02^↓	0		x		

**Notes.**

* These miRNAs were reported to be significantly deregulated by De Sarkar et al. (2014).

**Comparison with De Sarkar et al. (2014):** Among the sixteen miRNAs listed in Table 1, hsa-miR-133-3p, miR-206, miR-31-3p/5p, miR-1293, miR-7-5p, and miR-204-5p were identified as significantly deregulated in De Sarkar et al. (2014)’s analysis. De Sarkar et al.’s bioinformatics analyses showed that, except for miR-1293, the deregulation patterns of these miRNAs were concordant with previously published studies in OSCC. Although De Sarkar et al. (2014) used the BH method to control the FDR, they identified seven miRNAs instead of three because they imputed the missing values using a median-based imputation strategy. As discussed in ‘Missingness’, such imputations can increase detections by artificially deflating the sample variance. Figure S5.1 shows that the number of discoveries substantially increases for all four FDR-control methods if we impute the missing miRNA expressions with their median across the 18 patients. For Scheme (c) alone, the total number of detections rose from fourteen to twenty-seven after imputation by sample median, as shown in Table S5.1.

Among the sixteen miRNAs listed in Table 1, the nine miRNAs miR-1, miR-486-3p, miR-21-5p, miR-147b, miR-99a-3p, miR-455-3p, miR-1247-5p, miR-455-5p, and miR-486-5p were undetected by De Sarkar et al. (2014). For ease of reference, we will refer to these nine as “novel” miRNAs. Their relevance will be discussed in detail in ‘Relevance of the novel miRNAs’. As mentioned previously, De Sarkar et al. (2014) found that established cancer biomarkers hsa-miR-1 and miR-21-5p were downregulated and upregulated, respectively, in many samples, although neither was detected as significantly deregulated in their analysis (Heatmap 3). In particular, miR-1’s *p*-value was just above the BH-adjusted significance threshold in their study. Every group-adaptive BH method detected miR-1 to be significantly downregulated, regardless of the grouping scheme. TST-GBH detected miR-21-5p as significantly deregulated under all grouping schemes except the coarsest, Scheme (a). In a cluster analysis, De Sarkar et al. (2014) identified a subgroup of 13 patients with 30 deregulated miRNAs in cancer tissues (see Table 6 in De Sarkar et al., 2014). Except miR-486-3p/5p and miR-455-3p/5p, all novel miRNAs were members of this set of 30 miRNAs.

**Overlap with prior OSCC studies:** Most of the significantly deregulated miRNAs reported in Table 1, except miR-1293, miR-1247-5p, and miR-147b, have been mentioned in prior OSCC studies. Notably, hsa-miR-21-5p, miR-31, and miR-204-5p are among the most frequently reported diagnostic and prognostic markers across OSCC studies (Troiano et al., 2018; Ghosh, Pattatheyil & Roychoudhury, 2020; Min et al., 2015; Wong et al., 2016). In addition, miR-1 and miR-455-3p/5p have been highlighted in surveys and meta-analyses for their prognostic relevance (Troiano et al., 2018; Ghosh, Pattatheyil & Roychoudhury, 2020). Importantly, miR-21-5p, miR-204-5p, miR-1, and miR-455-3p/5p were identified only by the group-adaptive BH methods in our analysis (Table 1).

At the same time, our methods did not detect several miRNAs frequently cited for their potential prognostic value in OSCC, including miR-125b, miR-155, miR-16, miR-196a, miR-20a, and miR-32 (Min et al., 2015; Ghosh, Pattatheyil & Roychoudhury, 2020; Troiano et al., 2018). There are several possible reasons for this lack of overlap. First, most studies included in the cited reviews focused on tongue and floor-of-mouth cancers, which differ in clinical behavior and molecular features from GBSCC, the subtype predominant in the South Asian tobacco-chewing population (Pansare et al., 2019). In fact, many of these miRNAs, *e.g.*, miR-196a, miR-20a, and miR-32, were also absent in the dataset of De Sarkar et al. (2014). Second, the lack of overlap may also be due to the diminished statistical power, owing to the limited sample size of our data. A deeper investigation into the sources of this discrepancy is beyond the scope of this study. What is relevant here is that the group-adaptive BH methods succeeded in increasing detections relative to BH. Nonetheless, statistical methods can not recover signals that are not present in the data.

**Effect of data missingness:** Although we restricted our analysis to complete cases, missingness may still have influenced the performance of the GBH methods. Among the miRNAs in Table 1, miR-1293 showed the highest level of missingness, with 50% of observations missing. Evidence for its cancer-promoting role in the literature is limited. Table 1 includes five other miRNAs with missing values, miR-31-3p, miR-204-5p, miR-7-5p, miR-147b, and miR-1247-5p, but for each of these, the proportion of missing data was lower (10%). In this context, the magnitude of the ΔΔ*Ct* value serves as the effect size, as it measures the strength of differential expression between tumor and control tissues (Wasserstein & Lazar, 2016). Among the miRNAs with 10% missingness, the miRNAs with higher effect size, *e.g.*, miR-31-3p, miR-204-5p, and miR-7-5p, were previously implicated in OSCC (see De Sarkar et al., 2014 for details). By contrast, evidence supporting the observed deregulation of miR-147b and miR-1247-5p, which had relatively lower effect size, is relatively limited in OSCC. More details on these miRNAs can be found in ‘Relevance of the novel miRNAs’. Notably, miR-1293, miR-147b, and miR-1247-5p were mostly detected by TST-GBH (Table 1, Table S4.1). By contrast, SABHA generally did not detect any miRNAs with missing data to be significantly deregulated under the considered grouping schemes. The only exception was miR-31-3p, which was also identified by BH and is a well-established oncomiR in OSCC (Lin et al., 2022). Of the six detected miRNAs with missing values, miR-31-3p, miR-204-5p, miR-7-5p, and miR-1293 were also reported to be significantly deregulated in De Sarkar et al. (2014)’s analysis, which, we remind readers, used imputed data. We suspect that the small sample size amplified the adverse effects of missingness.

### Control study: random group assignment

To see if our position-based groups indeed helped with detection, as a small control study, we compared our results to the case where the miRNAs were assigned randomly to the groups. To this end, we restricted our attention to the groups in Scheme (c), where the groups are based on shared chromosome number, strands, and arms. Under random assignment, the groups remained identical to those of Scheme (c) in number and size, but the miRNAs were assigned to them randomly, not based on their position. Therefore, the groups were no longer biologically informative. The random assignment of the miRNAs to groups did not break the diseased-normal association, but broke the intra-group associations. The random assignments were carried out *N* = 1000 times, resulting in 1,000 replications. The group-adaptive BH methods were performed in each replication. The BH was not performed because it has no dependence on group assignments.

Under non-informative grouping, group-adaptive BH procedures degenerate to their corresponding single-group (“uni-group”) counterparts described in Section ‘Group-adaptive BH methods’ (Hu, Zhao & Zhou, 2010; Li & Barber, 2019). Accordingly, in the limit of replications (*N* → ∞), TST-GBH, LSL-GBH, and SABHA are expected to behave like TST-BH, LSL-BH, and Storey’s adaptive BH procedure, respectively. In our dataset, these three BH variants yield results identical to those of the standard BH procedure. Therefore, we will compare the average performance of the group-adaptive BH procedures under random grouping with that of BH.

#### Effect of random grouping on detections

With the exception of miR-135b-3p, the miRNAs frequently detected under random assignments (in more than 50% of replications) were also detected under Scheme (c) by at least one group-adaptive BH method. Table 2 provides the detection frequencies of these miRNAs. Table 3 provides summary statistics on the detections of each method across the 1,000 replications, and report their overlap with the three BH-detected miRNAs: miR-133a-3p, miR-31-3p, and miR-206. On average, the number of detections under random group assignments dropped to three for both SABHA and LSL-GBH. Moreover, most detections by LSL-GBH (about two out of three) and all detections by SABHA overlapped with the three BH miRNAs (Table 3). Table 1 shows that SABHA and LSL-GBH collectively detected cancer biomarkers such as miR-31-5p, miR-1, and miR-7-5p to be significantly deregulated under Scheme(c) (Lu et al., 2019; Safa et al., 2020; Jung et al., 2012). However, LSL-GBH and SABHA detected these miRNAs in less than two-third of the random assignments (Table 2). Therefore, LSL-GBH and SABHA’s detection of these miRNAs under Scheme (c) may be attributable to the additional power gained from position-based grouping.

**Table 2:** Detection of significantly deregulated miRNAs under 1,000 random reassignments of Scheme (c). Each replication randomly reassigns miRNAs to groups in Scheme (c), preserving group sizes and diseased–normal pairing but breaking intra-group associations. This table lists the miRNAs that were either (1) detected to be significantly deregulated under original (non-randomized) Scheme (c) by at least one of the four FDR-control methods or (2) identified as significantly deregulated in more than 50% of the random group assignments by at least one group-adaptive BH method. The percentages under TST (stands for TST-GBH), LSL (stands for LSL-GBH), and SABHA represent the proportion of 1,000 random assignments in which each miRNA was identified as significantly deregulated by the corresponding method. A cross x in these columns indicates that the miRNA was detected under the original (non-randomized) Scheme (c). Only hsa-miR-135b-3p (bolded) was not detected under the original Scheme (c). The “Chr.” column gives the chromosome, the “Str.” column indicates the chromosome strand, and the “arm” column specifies the arm. The “ΔΔCt” column provides the average ΔΔCt value for each miRNA across 18 patients. The “*p*-value” column shows raw *p*-values from paired t-tests. Rows are sorted by *p*-values, and the arrows following them denote whether the expression was upregulated (↑) or downregulated (↓) according to the sign of ΔΔCt. The “Missing pairs” column provides the number of missing pairs of observations for each miRNA.

**miRNA**	**Chr.**	**Str.**	**Arm**	ΔΔCt	***p*-value**	Missing pairs	**TST**	**LSL**	**SABHA**
hsa-miR-133a-3p^*^^,^^a^	18	–	q	6.7	5.32*E*^−05^↓	0	x (100%)	x (56%)	x (100%)
hsa-miR-31-3p^*^^,^^a^	9	–	p	−3.8	1.31*E*^−04^↑	2	x (100%)	x (51%)	x (100%)
hsa-miR-206^*^^,^^a^	6	+	p	6.0	1.97*E*^−04^↓	0	x (100%)	48%	x (100%)
hsa-miR-31-5p^*^	9	–	q	−3.4	6.18*E*^−04^↑	0	x (100%)	x (33%)	x (3%)
hsa-miR-204-5p^*^	9	–	q	4.6	8.81*E*^−04^↓	2	x (94%)	27%	3%
hsa-miR-1	18	–	q	5.2	9.38*E*^−04^↓	0	x (95%)	x (27%)	x (3%)
hsa-miR-7-5p^*^	15	+	q	−3.1	9.50*E*^−04^↑	2	x (94%)	x (27%)	3%
hsa-miR-1293^*^	12	–	q	−4.8	2.77*E*^−03^↑	9	x (76%)	9%	0%
hsa-miR-486-3p	8	–	p	2.4	2.81*E*^−03^↓	0	x (73%)	8%	0%
hsa-miR-21-5p	17	+	q	−2.2	3.67*E*^−03^↑	0	x (71%)	6%	0%
hsa-miR-147b	15	+	q	−2.1	5.52*E*^−03^↑	2	x (51%)	3%	0%
**hsa-miR-135b-3p**	**1**	**–**	**q**	**−2.0**	**5.73*E*^−03^↑**	**2**	**50%**	**3%**	**0%**
hsa-miR-99a-3p	21	+	q	2.8	5.83*E*^−03^↓	0	x (49%)	3%	0%
hsa-miR-1247-5p	14	–	q	2.4	8.93*E*^−03^↓	2	x (31%)	1%	0%
hsa-miR-486-5p	8	–	p	2.0	1.78*E*^−02^↓	0	x (8%)	0%	0%

**Notes.**

* These miRNAs were reported to be significantly deregulated by De Sarkar et al. (2014).

a These miRNAs were detected by BH to be significantly deregulated.

**Table 3:** Summary statistics of significantly deregulated miRNAs from 1,000 random assignments. The summary statistics are reported for group-adaptive methods TST-GBH (TST), LSL-GBH (LSL), and SABHA, listed under the column “Method.” (i) This table shows mean, median, and interquartile range (IQR) of the number of significantly deregulated miRNAs across 1,000 random assignments; (ii) This table shows mean, median, and IQR of the number of significantly deregulated miRNAs that overlap with the three BH-detected miRNAs (hsa-miR-133a-3p, miR-31-3p, and miR-206); and (iii) This table shows mean, median, and IQR of the number of detected miRNAs that overlap with the non-BH miRNAs. The non-BH miRNAs are the eleven miRNAs detected under Scheme (c) by group-adaptive BH methods but not by BH (see Table 1). In all tables, the summary statistics are calculated across the 1,000 replications.

(a) Distribution of detections (1,000 random assignments)			
Method	Mean	Median	IQR
TST	13.7	14	(12, 15)
LSL	3.1	3	(1, 5)
SABHA	3.1	3	(3, 3)

(b) Distribution of overlap for the 11 non-BH genes			
Method	Mean	Median	IQR
TST	9.5	10	(8, 11)
LSL	1.5	1	(0, 3)
SABHA	0.1	0	(0, 0)

(c) Distribution of overlap for the 3 BH genes			
Method	Mean	Median	IQR
TST	3.0	3	(3, 3)
LSL	1.5	2	(1, 2)
SABHA	3.0	3	(3, 3)

In contrast to LSL-GBH and SABHA, the average number of detections by TST-GBH did not decline substantially under random group assignment. On average, TST-GBH still identified 14 miRNAs as significantly deregulated (Table 3i). These detections always included the three miRNAs found by BH, along with miR-31-5p (Table 2). Among the remaining detections, about six miRNAs overlapped with those identified by this method under Scheme (c), while the rest varied randomly across samples. This pattern suggested that position-based grouping may have helped TST-GBH detect certain miRNAs, but the effect is less prominent than for SABHA and LSL-GBH. Overall, the control study provided stronger supporting evidence for SABHA and LSL-GBH in this dataset.

As mentioned earlier, miR-135b-3p (upregulated with ΔΔ*Ct* =  − 2.04) is the only new miRNA frequently detected across the replications. It is identified as significantly deregulated by TST-GBH in 50% of the replications (Table 2). It was also detected by TST-GBH under the finer grouping schemes derived from Scheme (c) when the maximum group size dropped below 6. miR-135b is frequently reported as upregulated in several cancers, including OSCC, and has been speculated to act as a promoter of OSCC (Olasz et al., 2015; Shao et al., 2025). Apart from miR-135b-3p, 43 additional miRNAs were detected in at least one of the 1,000 replications. However, no method detected them to be significantly deregulated in more than 40% of the replications.

### Relevance of the novel miRNAs

Although it would have been desirable to experimentally validate the pathways downstream of the novel miRNAs, such analyses were beyond the scope of the current study because of the exhaustion of biospecimens used by De Sarkar et al. (2014). However, several other functional works provided supporting evidence for the role of the “novel” miRNAs in carcinogenesis. Since 10 of the 12 cancer tissues assessed by Singh et al. (2017) overlap with the cohort studied in De Sarkar et al. (2014), their whole transcriptome analysis allowed for correlative analysis. We further compared our results with previously published onco-miRNAome studies.

Singh et al. (2017) assessed the differential expression of the genes (measured as mRNA transcripts) using EdgeR. EdgeR uses the BH method as a default choice for multiple-testing correction. Singh et al. (2017) considered a gene to be significantly deregulated if it showed at least 1.5-fold change and an FDR-adjusted *p*-value <0.05. Similar to us, they avoided imputations, and removed genes with low or inconsistent expression value before analysis. Eight of the nine novel miRNAs had some of their known target genes significantly deregulated in the opposite direction of the miRNA’s deregulation pattern in Singh et al. (2017)’s analysis. Table 4 lists these genes with their average fold changes, along with source articles reporting these genes as targets of the corresponding miRNAs. More details on these miRNAs and the target genes are provided in Supplement S6. miR-147b was the only novel miRNA without any significantly deregulated target gene in Singh et al. (2017)’s analysis. To our knowledge, miR-147b has not been previously characterized in OSCC. However, it has been implicated as an oncogenic miRNA in other cancers, such as lung cancer, which is consistent with its observed upregulation in our study (Zhang et al., 2019; Wang et al., 2020; Meng et al., 2013; Cui et al., 2015).

**Table 4:** Target genes of the newly discovered miRNAs with significantly deregulated expressions, as identified by Singh et al. (2017). All genes in the “Deregulated target genes in SCC” column, except FAM101B, are validated targets of the corresponding miRNA in the squamous cell carcinoma (SCC) subtype indicated next to each gene. Source references are provided in the adjacent “References” column. In addition, Singh et al. (2017) also reported genes that have been described as miRNA targets in other cancers but not specifically in SCC. These are listed in a separate column with their corresponding references in the adjacent “References” column. All genes reported in this table were significantly deregulated in Singh et al. (2017)’s whole transcriptome analysis in the opposite direction of the miRNA deregulation pattern. For each gene, the average fold change and direction of deregulation are shown in parentheses next to the gene name. The deregulation patterns of the corresponding miRNAs are also provided.

**miRNA**	Deregulated target genes in SCC	**References**	Deregulated target genes in other cancers	**References**
hsa-miR-1 (↓)	SNAI2 (↑, 2.2, OSCC); PNP (↑, 1.7, MSSCC); FAM101B^a^ (↑, 1.6, HNSCC)	Peng et al. (2017), Nohata et al. (2011a) and Nohata et al. (2011b)	PDE7A (↑, 1.9); CXCR4 (↑, 4.1); ZNF281 (↑, 1.6)	Yamamoto et al. (2015), Khan et al. (2023) and Cormier & Pollock (2004)
hsa-miR-21-5p (↑)	PDCD4 (↓, 2.9, OSCC)	Arslan Bozdag et al. (2024)	MEF2C (↓, 1.7); TIMP3 (↓, 2.7); PPARA (↓, 1.7); RECK (↓, 2.5); SPRY1 (↓, 2.0); SPRY2 (↓, 2.9); THRB (↓, 3.0)	Buscaglia & Li (2011)
hsa-miR-486-3p (↓)	N/A	N/A	MARCH1^*^ (↑, 1.9); FLNA (↑, 2.1)	Yang et al. (2023) and ElKhouly, Youness & Gad (2020)
hsa-miR-486-5p (↓)	N/A	N/A	NRP2^*^ (↑, 2.2); KIAA1199 (↑, 3.0)	Liu et al. (2022a) and Jiao et al. (2019)
hsa-miR-99a-3p (↓)	BCAT1 (↑, 3.2, HNSCC); MTHFD2 (↑, 1.5, HNSCC); RAC2 (↑, 3.5, HNSCC)	Okada et al. (2019) and Wei et al. (2019)	NCAPG^*^ (↑, 1.7); RRM2 (↑, 2.7)	Arai et al. (2018), Osako et al. (2019) and Okada et al. (2019)
hsa-miR-1247-5p (↓)	N/A	N/A	STMN1 (↑, 1.5)	Zhang et al. (2016)
hsa-miR-455-3p (↑)	ELF3 (↓, 4.8, OSCC)	Liu et al. (2022b)	N/A	N/A
hsa-miR-455-5p (↑)	PTPRS (↓, 2.9, OSCC)	Wu et al. (2021)	PDCD4^*^ (↓, 2.9)	Aili, Paizula & Ayoufu (2018)

**Notes.**

Abbreviations SCC squamous cell carcinoma MSSCC maxillary sinus SCC HNSCC head and neck SCC

* Associated with OSCC but, to our knowledge, not validated as direct targets in OSCC (see ‘Relevance of the novel miRNAs’).

a Nohata et al. (2011b) found that FAM101B was significantly downregulated in HNSCC cells following miR-1 transfection and contained a predicted miR-1 target site. However, to our knowledge, FAM101B has not been validated as a direct target of miR-1.

## Discussion

Biological studies often involve testing many hypotheses simultaneously, increasing the risk of false discoveries. Controlling the FDR allows investigators to strike a balance between the detection of true positive findings and minimizing the likelihood of false discoveries. The BH method is arguably the most widely used approach for FDR control in bioinformatics (Haynes, 2013). However, it compromises statistical power because it remains agnostic to additional structure among the hypotheses (Hu, Zhao & Zhou, 2010; Li & Barber, 2019). This limitation is especially problematic in small-sample studies where the number of hypotheses far exceeds the sample size, such as the oral cancer miRNA dataset reanalyzed here. In the dataset’s original report, De Sarkar et al. (2014) observed that OSCC-related miRNAs such as miR-1 and miR-21-5p failed to meet the FDR cutoff with the stringent BH method, although exploratory analyses suggested their deregulation. Moreover, a follow-up study by Singh et al. (2017) found that several target genes of these miRNAs were significantly deregulated in a cohort largely overlapping with De Sarkar et al. (2014)’s.

Although various types of structural information can be leveraged in the FDR control step, here we focused specifically on group structure. Group-adaptive BH methods are modifications of the standard BH method that use information on group structure to improve statistical power. Because De Sarkar et al. (2014)’s data were generated under rigorous analysis techniques, we used it as a test case to examine the potential advantages of these methods over BH. In this proof-of-principle study, our aim was not to identify new biomarkers, but to illustrate how incorporating simple biological structure, here, positional groupings of miRNAs, can influence the outcome of FDR control.

This study considered three group-adaptive BH methods, LSL-GBH, TST-GBH, and SABHA, chosen for their simplicity, feasibility in small-sample settings, and suitability for demonstrating how group information can be incorporated into the FDR control step. We compared several positional grouping schemes, in which miRNAs were clustered by chromosomal location with increasing levels of refinement. Across methods, the number of detections generally tended to increase as grouping schemes became finer. SABHA was the most conservative and showed the least sensitivity to changes in grouping schemes. In contrast, TST-GBH was the most liberal and most sensitive to changes in group sizes, consistent with earlier reports of its behavior (Sarkar & Zhao, 2022). In the final results, we considered grouping schemes with moderately sized groups, where all methods were relatively stable.

The group-adaptive BH methods collectively detected several miRNAs that were not detected as significantly deregulated by BH, including miR-1 and miR-21-5p. Most of these miRNAs have been previously associated with OSCC with deregulation patterns consistent with our study (‘Relevance of the novel miRNAs’). These miRNAs had several targets deregulated in the opposite direction (of the miRNA expression) in the follow-up transcriptome analysis of Singh et al. (2017), providing further support for their biological relevance. Despite TST-GBH being more liberal, its list of detections contained only two miRNAs without prior implications in OSCC, namely, miR-147b and miR-1293. Both miRNAs, especially miR-1293, had relatively high level of missingness. By contrast, all detections of LSL-GBH and SABHA, including those with missing values, had previous implications in OSCC. Taken together, in this dataset, group-adaptive BH methods showed potential for detecting signals missed by BH, although sensitivity to grouping and missingness varied across methods.

We recognize that grouping of miRNAs based on broad spatial information is overly simplistic. Groupings could also be defined by other forms of functional or genetic relatedness, such as ancestral similarity. However, exploring these alternatives was beyond the scope of this proof-of-principle analysis. While positional information does not capture all dimensions of functional relatedness, it provides an interpretable grouping strategy appropriate for the limited sample size of our dataset. Our control study with random group assignments suggested that the additional detections by LSL-GBH and SABHA may be attributable to the incorporation of positional grouping, indicating that even basic structural information has the potential to improve detections.

Group-adaptive BH methods have been applied across a wide range of sample sizes (Hu, Zhao & Zhou, 2010; Li & Barber, 2019), with applications in the literature including datasets with sample sizes even in the tens (Sankaran & Holmes, 2014; Li & Barber, 2019; Liu et al., 2015). Since these procedures operate directly on *p*-values, they do not pose additional computational challenges as the sample size increases. Likewise, these methods do not require estimation of model parameters using the individual observations, and thus do not suffer from numerical instability in small-sample settings. That said, the potential advantage of group-adaptive BH methods over the standard BH method will be more prominent in small to moderate sample size regimes, where individual tests tend to have lower power and the BH procedure can be overly conservative (Hu, Zhao & Zhou, 2010; Li & Barber, 2019).

Similarly, there is no hard lower or upper cutoff on the total number of hypotheses *p*. SABHA has been validated on synthetic datasets with a few hundred hypotheses and applied to problems with over 22,000 tests (Li & Barber, 2019), while the GBH methods have been used in settings ranging from tens of hypotheses (cf. applications in Sankaran & Holmes, 2014) to high-throughput DNA methylation studies involving over five million hypotheses (Zhang et al., 2013). When the total number of hypotheses is very small (*e.g.*, *p* < 100), the standard BH procedure may already be adequate.

However, the size of the groups is an important issue because, as we have seen, the number of detections is sensitive to the grouping scheme (‘Results’). Groups with a large number of hypotheses may be preferable because they lead to better acquisition of the group-level information (Hu, Zhao & Zhou, 2010; Li & Barber, 2019; Sarkar & Zhao, 2022). However, if the number of groups is chosen to be too small in order to preserve large group sizes, it will also be a problem. In this case, the resulting methods will behave similarly to their non-grouped counterparts—namely, Storey’s adaptive BH, TST-BH, or LSL-BH (Hu, Zhao & Zhou, 2010; Li & Barber, 2019)—thereby forfeiting the potential gains from incorporating group structure. Naturally occurring groups are generally preferred for group-adaptive methods, but their sizes can be non-uniform (Sarkar & Zhao, 2022). Application of group-adaptive BH methods often involves examples where groups are small or many groups have size below five (Sankaran & Holmes, 2014; Sarkar & Zhao, 2022; Nandi, Sarkar & Chen, 2021). In our analysis, we explored multiple grouping schemes and observed that the GBH methods become too loose when the maximum group size dropped below ten (‘Results’). We therefore recommend selecting the grouping scheme carefully, for example, using an elbow method based on the number of discoveries, as illustrated in ‘Results’.

Alternative grouping strategies, such as ontology-based assignments (Liu et al., 2015) or data-driven clustering methods, *e.g.*, k-means, tight clustering (Karmakar et al., 2019), WGCNA (Horvath, 2011), may also be useful. The data-dependent clustering approaches, however, must be applied with caution, as recent work has shown that reusing the same data for both clustering and hypothesis testing can inflate false discoveries (Lähnemann et al., 2020; Gao, Bien & Witten, 2020). We remind the readers that independence between groups is not required. Group-adaptive BH methods are generally most advantageous when most hypotheses are null and the intra-group association is higher than inter-group association (Hu, Zhao & Zhou, 2010; Chu et al., 2017). Existing literature suggests the first condition is likely in miRNAome data (Osan et al., 2021), and our analyses in ‘Intra- and inter-group association’ indicated that the second is also plausible for our location-based grouping schemes.

In addition to the group-adaptive BH methods studied here, other approaches exist for incorporating group structure into FDR control, such as independent hypothesis weighting (Ignatiadis et al., 2016), q-values (Storey, 2002), and knock-off (Sesia et al., 2021). Our intent was not to benchmark all available approaches or to claim superiority of the group-adaptive BH methods. Rather, in this proof-of-concept analysis, we aimed to demonstrate that even simple methods that exploit external grouping information can make discoveries that are missed by the standard BH procedure. The promising performance observed here suggests that more sophisticated approaches may also be effective, but this remains to be evaluated systematically in future work. The choice of method should ultimately depend on the scale and characteristics of the dataset.

The replicability of our results to future datasets of different scales and types remains a promising avenue for further investigation. Although our analysis was based on a miRNAome dataset, the strategy of incorporating group information into the FDR control step can be applied to any multiple testing scenario. Of course, controlled future analyses are needed to test such generalizability. In particular, extrapolation to larger datasets generated by high-throughput sequencing technologies remains to be tested. Another setting of particular interest comprises studies of treatment-emergent cancer subtypes, which often have small sample sizes similar to our study (Aggarwal et al., 2019; Clermont et al., 2019). These types of cancer arise as a result of treatment-related factors, potentially as a consequence of the treatment itself or because of the underlying conditions being treated. The genes responsible for such cancer subtypes are still not fully elucidated. Future studies aiming to unveil these genetic factors will necessitate FDR control because of multiple hypothesis testing.

## Conclusion

The conservativeness of the BH method can limit scientific discoveries, especially in studies where the sample size is small but hundreds of hypotheses need to be tested simultaneously. Using the miRNAome dataset of De Sarkar et al. (2014), we demonstrated that recent modifications of the BH method that incorporate group structure can offer advantages in such settings. Incorporating even simple group information, such as positional grouping among miRNAs, appeared to contribute to improved detections, although sensitivity to grouping varied across methods. While our study was limited to a single moderately scaled dataset, the results highlight the potential of group-adaptive BH methods for small-sample studies where statistical power is a concern. Future work should assess these methods across datasets of varying scales and designs and explore alternative grouping strategies and more advanced FDR control procedures to establish their broader applicability.

##  Supplemental Information

10.7717/peerj.21257/supp-1 Supplemental Information 1 Additional discussion, figures, and tables

## Appendix

### Simple random effect model to quantify the inter-group and intra-group correlation

To formalize the setup, we will introduce some new notation. Denote by {*Y*_*ij*_:}j=1{, …, 522} the ΔΔ*Ct* values of the *j*th miRNA of the *i*th subject/ patient. Let *g*(*j*) be the group indicator of the *j*th miRNA, *e.g.*, if the *j*th miRNA belongs to the first group, then *g*(*j*) = 1. The group index *g*(*j*) takes value between 1 and 67.

We will posit a random effect model (Searle, Casella & McCulloch, 2009) on the ΔΔ*Ct* values as

\begin{eqnarray*}{Y}_{ij}=\mu +{S}_{i}+{G}_{ig(j)}+{E}_{ij}, \end{eqnarray*}

where µis the overall mean, *S*_*i*_ is the random effect of the ith patient, *G*_*ig*(*j*)_ is the random effect of the *g*(*j*)th group for the *i*th patient, and *E*_*ij*_ is the random error for the *i*th subject and the *j*th miRNA. The random error can represent independent measurement errors or other underlying biological factors. We let the collections of random variables {*S*_*i*_:*i* = 1, …, 18}, {*G*_*ig*(*j*)_:1 ≤ *i* ≤ 18,  1 ≤ *j* ≤ 522}, and {*E*_*ij*_:1 ≤ *i*, *j* ≤ 522} to be independent of each other. We will also assume that the *S*_*i*_’s are identically distributed as $N(0,{\sigma }_{s}^{2})$, the *G*_*ig*(*j*)_’s are identically distributed as $N(0,{\sigma }_{g}^{2})$, and the *E*_*ij*_’s are identically distributed as $N(0,{\sigma }_{e}^{2})$, where ${\sigma }_{s}^{2}$, ${\sigma }_{g}^{2}$, and ${\sigma }_{e}^{2}$ are unknown positive numbers.

Under the above model, the correlation between the ΔΔ*Ct* values of two miRNAs belonging to two different groups within the same subject is constant. The above correlation will thus be a natural candidate for the inter-group correlation. Straight-forward algebra shows that the inter-group correlation equals ${\sigma }_{s}^{2}/({\sigma }_{s}^{2}+{\sigma }_{g}^{2}+{\sigma }_{e}^{2})$. Likewise, under our random effect model, the ΔΔ*Ct* values of two miRNAs belonging to the same group within the same subject have identical correlations across the subjects and the miRNAs—this correlation will be a natural candidate for the intra-group correlation. The intra-group correlation is given by $({\sigma }_{s}^{2}+{\sigma }_{g}^{2})/({\sigma }_{s}^{2}+{\sigma }_{g}^{2}+{\sigma }_{e}^{2})$. We used the R package lme4 to fit the model and obtain estimates of the intra- and inter- group correlations.

## Additional Information and Declarations

### Data Availability

The following information was supplied regarding data availability: The data is available in the Supplementary Files of De Sarkar et al. (2014): De Sarkar N, Roy R, Mitra JK, Ghose S, Chakraborty A, Paul RR, et al. (2014) A Quest for miRNA Bio-Marker: A Track Back Approach from Gingivo Buccal Cancer to Two Different Types of Precancers. PLoS ONE 9(8): e104839. https://doi.org/10.1371/journal.pone.0104839. The code is available at Github: - https://github.com/nilanjanalaha/GABH.git
- nilanjanalaha/GABH: GABHv1 (code). Zenodo. https://doi.org/10.5281/zenodo.18748238.
