# Supplementary material for: False discovery rate control for grouped hypotheses: application to miRNAome data
Source: PeerJ. 2026 May 25;14:e21257. doi: 10.7717/peerj.21257 (PMC13218340; doi:10.7717/peerj.21257)
Supplement: Supplemental Information 1 [file peerj-14-21257-s001.pdf]

Supplement to “False Discovery Rate Control for Grouped Hypotheses: Application to miRNAome Data”

Nilanjana Laha[1], Salil Koner[2], Austin Labowitz[3], and Navonil De Sarkar[4]

[1,3]**Department of Statistics, Texas A&M, College Station, Texas, USA**
[2]**Department of Statistics, University of California, Riverside, CA, USA.**
[4]**Department of Pathology, Medical College of Wisconsin, Milwaukee, Wisconsin, USA**

Corresponding author:
Nilanjana Laha[1]

Email address: nlaha@tamu.edu

ABSTRACT

This document contains supplementary material and does not include an abstract.

S1 MIRNAOME DATA GENERATION

The miRNA profiling was performed using the TaqMan Low-Density Array (TLDA), a high-throughput qPCR assay suited for quantifying standard and low-expressing miRNAs in biospecimens (Wang et al., 2011). RNA was isolated using the mirVana kit (Life Technologies, USA), and only samples with RNA integrity number (RIN) ≥ 6.9 were included. Ct, ΔCt, and ΔΔCt values were computed using the SDS and DataAssist software packages (Life Technologies, USA). The definitions are as follows. Ct is the cycle number at which the PCR product reaches a pre-specified fluorescent detection threshold. ΔCt is the difference between the Ct of a miRNA and the geometric mean Ct of the three most stable endogenous control miRNAs in that tissue. ΔΔCt is the difference between a miRNA’s ΔCt in tumor and in matched control tissue. The raw data were normalized and trimmed following the standard TLDA data analysis protocol. Further details on the data collection methods are available in (De Sarkar et al., 2014).

S2 MISSINGNESS

Figure S2.1 shows that the number of missing miRNA pairs can range from 12 to 232 across the eighteen patients. Only two patients exhibit more than 100 missing pairs, while the majority, 70% of the patients, have fewer than 50 missing pairs. This histogram in Figure S2.2 indicates that a significant proportion, approximately 59.77%, of the miRNAs possess at least one pair of missing observations. However, most of these miRNAs (about 48.76% of them) have at most two missing pairs of observations. Nevertheless, there are fifteen miRNAs with 50% missing observations each.

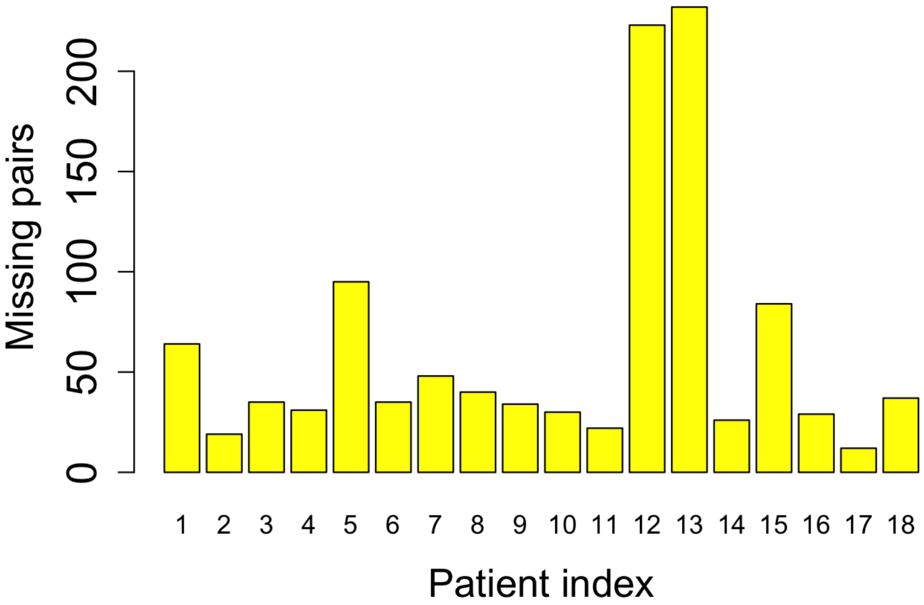

Figure S2.1. Barplot of the number of missing pairs of miRNAs across patients. Each bar corresponds to a patient, and the y-axis corresponds to the number of missing (tumor-control) pairs of miRNAs corresponding to each patient.

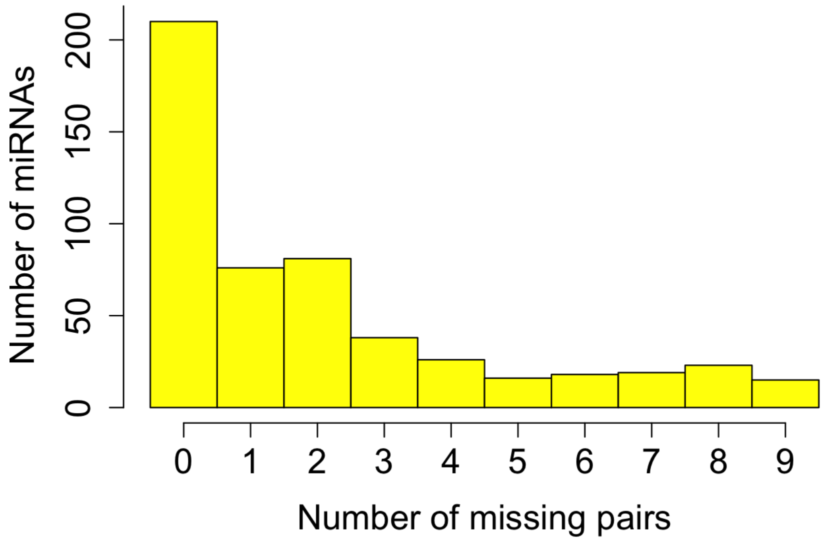

Figure S2.2. Histogram of the number of missing pairs of miRNAs. Here we considered the 522 miRNAs from our analysis. The x-axis corresponds to the number of missing pairs, and the y-axis corresponds to the number of miRNAs with that many missing pairs.

S3 PLOTS AND TABLES RELATED TO THE GROUPS

Table S3.1. Table of group composition for Scheme (c). This table lists the size, chromosome number, strand, and arm for each group in Scheme (c). Here, size refers to the number of miRNAs in a group. The groups are ordered according to their Chromosome number. Some groups are situated on both p and q arms because single-miRNA groups were fused with the adjacent group to make a larger group.

Group number	Size	Chromosome number	Strand	Arm
1	9	1	-	p
2	19	1	-	q
3	10	1	+	p
4	3	1	+	q
5	3	2	-	p
6	3	2	-	q
7	8	2	+	q
8	8	3	-	p
9	2	3	-	q
10	4	3	+	p
11	10	3	+	q
12	3	4	-	p
13	6	4	-	q
14	4	4	+	p
15	5	4	+	q
16	2	5	-	p
17	9	5	-	q
18	7	5	+	q
19	4	6	-	p, q
20	2	6	+	p
21	6	7	-	p
22	17	7	-	q
23	2	7	+	p
24	7	7	+	q
25	5	8	-	p
26	8	8	-	q
27	3	8	+	p, q
28	3	9	-	p
29	4	9	-	q
30	14	9	+	p, q
31	2	10	-	p
32	4	10	-	q
33	2	10	+	p
34	4	10	+	q
35	4	11	-	p
36	11	11	-	q
37	6	11	+	p, q
38	8	12	-	p, q
39	5	12	+	p
40	8	12	+	q
41	5	13	-	q
42	13	13	+	q
43	4	14	-	q
44	55	14	+	q

Continued on next page

Table S3.1 – continued from previous page

Group number	Size	Chromosome number	Strand	Arm
45	9	15	-	q
46	7	15	+	q
47	2	16	-	p, q
48	5	16	+	p
49	3	16	+	q
50	13	17	-	p
51	17	17	-	q
52	2	17	+	p
53	5	17	+	q
54	3	18	-	q
55	8	19	-	p
56	3	19	-	q
57	5	19	+	p
58	37	19	+	q
59	3	20	-	p, q
60	7	20	+	p
61	6	21	+	q
62	4	22	-	q
63	10	22	+	q
64	6	X	-	p
65	32	X	-	q
66	9	X	+	p
67	5	X	+	q

Sample	Site	Age	Sex	Histopathological observation
S1	Right Buccal Mucosa (Cheek)	39	M	Well diff
S2	Cheek	55	M	Well diff
S5	Right Cheek	65	F	Mod diff
S6	Right Lower Buccal Mucosa	60	F	Well diff
S7	Cheek	40	M	Well diff
S8	Cheek	45	M	Well diff
S11	Gingiva	52	M	Mod diff
S12	Right Gingiva	45	F	Well diff
S13	Left Buccal Mucosa (Cheek)	60	F	Well diff
S15	Right Buccal Mucosa (Cheek)	45	F	Well diff
S16	Left Cheek	40	F	Well diff
S17	Right Retro-molar Region	51	M	Well diff
S19	Left Buccal Vestibule	68	F	Well diff
S20	Left Buccal Mucosa (Cheek)	66	M	Well diff
S21	Left Buccal Mucosa (Cheek)	45	M	Well diff
S26	Gingiva	40	M	Well diff
S27	Cheek	55	F	Well diff
S24	Cheek	80	M	Well diff

Table S3.2. Demography of 18 cancer patients and their tumor differentiation status. This table lists the sample ID, tumor site, patient age, sex, and histopathological observation, reproduced from De Sarkar et al. (2014). All patients included in this demography had tobacco habits. Tumor differentiation denotes histological grading based on resemblance to normal squamous epithelium (well differentiated vs. moderately differentiated).

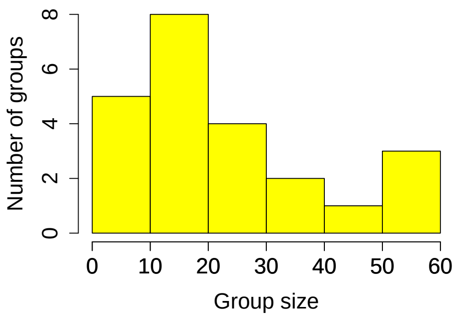

(i) Scheme (a). Max size: 59, min: 3.

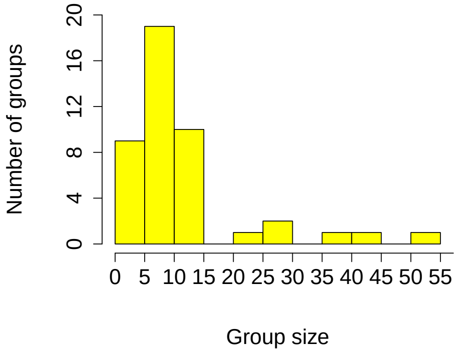

(ii) Scheme (b). Max size: 55, min: 2.

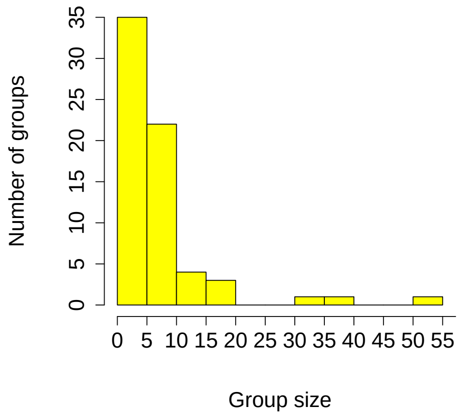

(iii) Scheme (c). Max size: 55, min: 2.

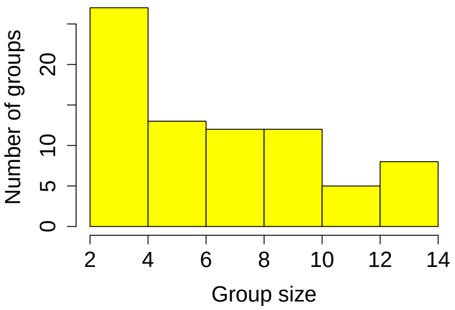

(iv) Scheme with $k = 15$. Max size: 15, min: 2.
S3.1iv

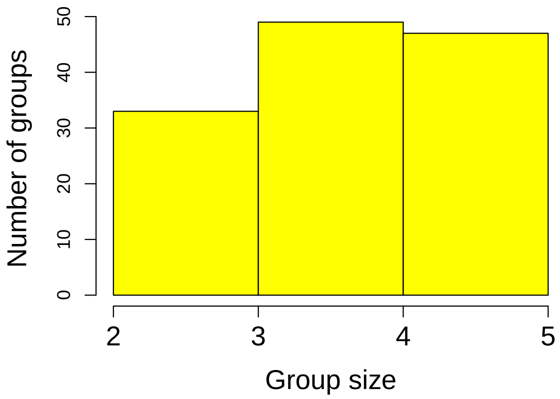

(v) Scheme with $k = 5$. Max size: 5, min: 2.

Figure S3.1. Histograms of group sizes under different grouping schemes. Group size refers to the number of miRNAs in a group. Panels (i)–(iii) correspond to Grouping Schemes (a), (b), and (c), respectively. Panels (iv) and (v) correspond to the finer grouping schemes in Section 2.4 with $k = 15$ and 5, respectively. The x-axis shows group size, and the y-axis shows the number of groups. For each scheme, “max” and “min” indicate the largest and smallest group sizes.

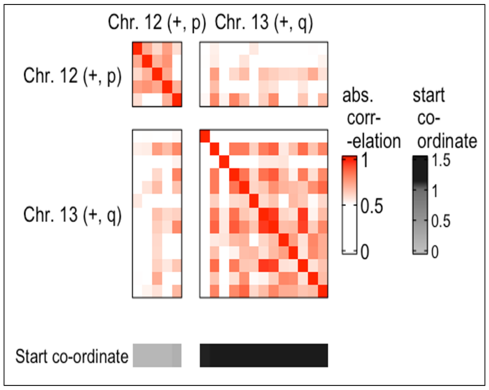

(i) Position-based groups

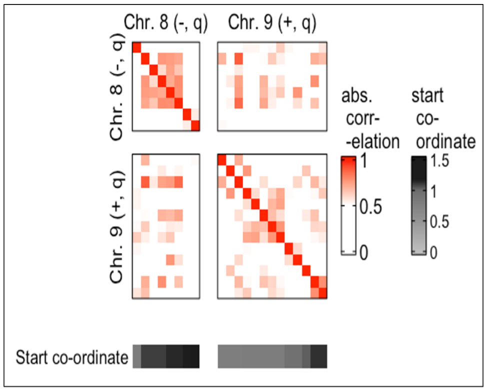

(ii) Position-based groups

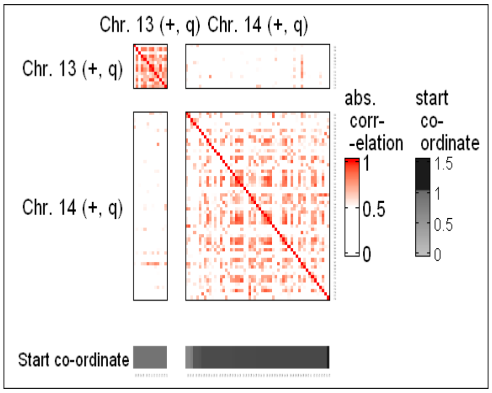

(iii) Position-based groups

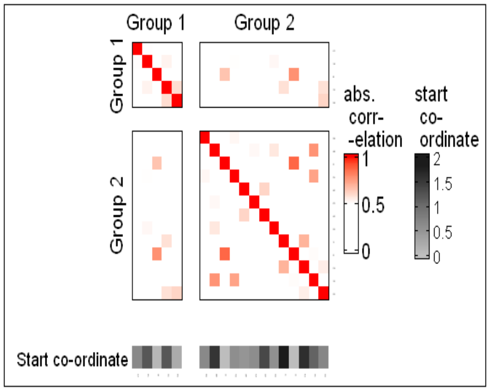

(iv) Pseudo groups with randomly picked miRNAs

Figure S3.2. Heatmap of the absolute correlations between the ΔΔCt values of the miRNAs from three groups. (i) The first group contains miRNAs from the q arm of the positive strand of chromosome 9 and the second group contains miRNAs from the q arm of the negative strand of chromosome 8. (ii) The first group corresponds to the p arm of the positive strand of chromosome 12 and the second group corresponds to the miRNAs on the q arm of the positive strand of chromosome 13. (iii) The first group corresponds to the q arm of the positive strand of chromosome 13 and the second group corresponds to the miRNAs on the q arm of the positive strand of chromosome 14. (iv) The pseudo-groups have the same number of miRNAs as the groups in panel (ii), but the miRNAs were picked randomly. The diagonal blocks provide the heatmaps of the absolute correlation between the ΔΔCt values of miRNAs within the same group, whereas the off-diagonal blocks showcase the absolute correlation between ΔΔCt values of miRNAs from different groups. Only absolute correlations above 0.5 are presented in the heatmaps. The sidebars below the heatmaps provide the start coordinates of the miRNAs. For panels (i)–(iii), the miRNAs are ordered based on their start coordinates on the chromosome, ensuring that adjacent miRNAs are positioned next to each other in the heatmap.

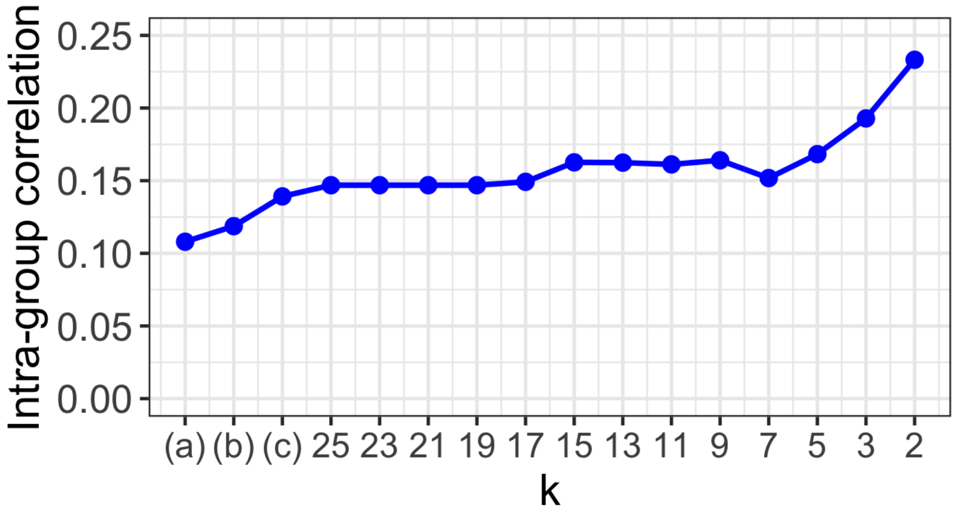

(i) Intra-group correlation vs k

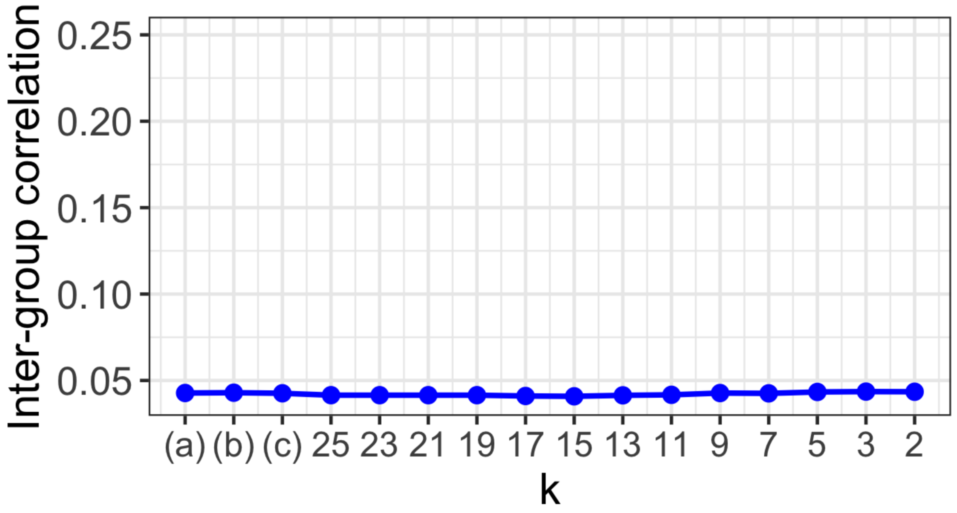

(ii) Inter-group correlations vs k

Figure S3.3. Plot of intra- and inter-group correlations. The x-axis corresponds to different grouping schemes, and the y-axis represents the correlation estimates for each scheme. For each grouping scheme, the intra- and inter-group correlations are computed according to the simple random effect model discussed in Appendix 5. The grouping schemes (a), (b), and (c) are described in Section 2.4. The schemes corresponding to $k \in \{25, \dots, 2\}$ are finer partitions obtained by further splitting the groups in Scheme (c), as detailed in Section 2.4. Here, apart from the k's used in the main manuscript, i.e., $k \in \{25, 23, \dots, 5\}$, we also consider $k = 2$ and 3 to highlight how the intra-group correlation increases in smaller groups.

S4 TABLES AND FIGURES CORRESPONDING TO SECTION 3

miRNA	Chr.	Strand	Arm	ΔΔCt	p-value	Missing pairs	BH	TST	LSL	SABHA
hsa-miR-133a-3p*	18	-	q	6.7	$5.32E^{-05}\downarrow$	0	x	x	x	x
hsa-miR-31-3p*	9	-	p	-3.8	$1.31E^{-04}\uparrow$	2	x	x	x	x
hsa-miR-206*	6	+	p	6.0	$1.97E^{-04}\downarrow$	0	x	x	(a)	x
hsa-miR-31-5p*	9	-	q	-3.4	$6.18E^{-04}\uparrow$	0		x	(a)	
hsa-miR-204-5p*	9	-	q	4.6	$8.81E^{-04}\downarrow$	2		x	x	
hsa-miR-1	18	-	q	5.2	$9.38E^{-04}\downarrow$	0		x	x	x
hsa-miR-7-5p*	15	+	q	-3.1	$9.50E^{-04}\uparrow$	2		x	x	
hsa-miR-1293*	12	-	q	-4.8	$2.77E^{-03}\uparrow$	9		(b)		
hsa-miR-486-3p	8	-	p	2.4	$2.81E^{-03}\downarrow$	0		x		
hsa-miR-21-5p	17	+	q	-2.2	$3.67E^{-03}\uparrow$	0		(b)		
hsa-miR-147b	15	+	q	-2.1	$5.52E^{-03}\uparrow$	2		x		
hsa-miR-99a-3p	21	+	q	2.8	$5.83E^{-03}\downarrow$	0		x		
hsa-miR-455-5p	9	+	q	-1.4	$5.98E^{-03}\uparrow$	0		(a)		
hsa-miR-1247-5p	14	-	q	2.4	$8.93E^{-03}\downarrow$	2		(b)		

Table S4.1. Significantly deregulated miRNAs for grouping Schemes (a) and (b). The listed miRNAs were detected as significantly deregulated (5% significance level) by BH, TST-GBH (TST), LSL-GBH (LSL), or SABHA under either Scheme (a) or Scheme (b). If a method detected a miRNA with both grouping schemes, the corresponding row is marked by x. Otherwise, the row marks the grouping scheme under which the corresponding miRNA were detected. The "Chr." column gives the chromosome, the "Str." column indicates the chromosome strand, and the "arm" column specifies the arm. The "ΔΔCt" column provides the average ΔΔCt value for each miRNA across 18 patients. The "p-value" column shows raw p-values from paired t-tests. Rows are sorted by p-values, and the arrows following them denote whether the expression was upregulated (↑) or downregulated (↓) according to the sign of ΔΔCt. The "Missing pairs" column provides the number of missing pairs of observations for each miRNA. $*$ These miRNAs were reported to be significantly deregulated by De Sarkar et al. (2014).

S5 RESULTS AFTER MEDIAN IMPUTATION

Figure S5.1 shows the number of significantly deregulated miRNAs across grouping schemes after median imputation, and Table S5.1 reports the significantly deregulated miRNAs detected under grouping Scheme (c). A comparison between Figures S5.1 and 2 shows that the number of detections increased substantially across all methods and grouping schemes after imputation. Table S5.2 summarizes the increase in the number of detections for all four FDR control methods across representative grouping schemes. The magnitude of the increase, ranging roughly between seven and thirteen additional detections, is broadly comparable across all four methods.

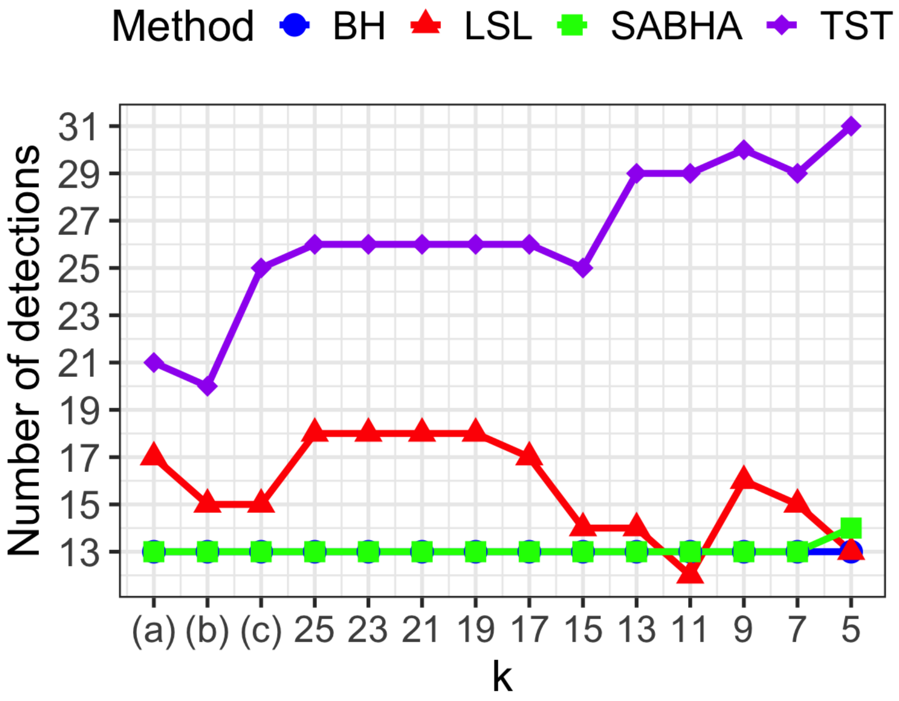

Figure S5.1. Plot of the number of detections by each FDR control method vs grouping scheme for the median-imputed data. The methods compared were BH, TST-GBH (abbreviated as TST), LSL-GBH (abbreviated as LSL), and SABHA. The grouping schemes (a), (b), and (c) are described in Section 2.4. The schemes corresponding to $k = 5, \ldots, 25$ are finer partitions obtained by further splitting the groups in Scheme (c), as detailed in Section 2.4. The y-axis shows the total number of detections from each grouping scheme.

MiRNA	Chr	Strand	Arm	ΔΔCt	p-value	Missing pairs	BH	TST	LSL	SABHA
hsa-miR-1293	12	-	q	-4.8	1.51E-06 ↑	9	x	x		x
hsa-miR-31-3p	9	-	p	-3.8	2.00E-05 ↑	2	x	x	x	x
hsa-miR-548k†	11	+	p	-2.7	3.86E-05 ↑	8	x	x		x
hsa-miR-133a-3p	18	-	q	6.7	5.32E-05 ↓	0	x	x	x	x
hsa-miR-206	6	+	p	6.0	1.97E-04 ↓	0	x	x		x
hsa-miR-204-5p	9	-	q	4.6	2.19E-04 ↓	2	x	x		x
hsa-miR-7-5p	15	+	q	-3.1	2.80E-04 ↑	2	x	x	x	x
hsa-miR-891a-5p†	X	-	q	4.4	2.84E-04 ↓	7	x	x	x	x
hsa-miR-504-5p†	X	-	q	4.7	6.00E-04 ↓	6	x	x	x	x
hsa-miR-31-5p	9	-	p	3.4	6.18E-04 ↑	0	x	x	x	x
hsa-miR-133b†	6	+	p	2.7	6.80E-04 ↓	8	x	x		x
hsa-miR-508-3p†	X	-	q	4.0	7.91E-04 ↓	6	x	x	x	x
hsa-miR-1	18	-	q	5.2	9.38E-04 ↓	0	x	x	x	x
hsa-miR-135b-3p†	1	-	q	-2.2	2.42E-03 ↑	2		x		
hsa-miR-200b-5p†	1	+	p	-3.5	2.66E-03 ↑	3		x		
hsa-miR-486-3p	8	-	p	2.4	2.81E-03 ↓	0		x	x	
hsa-miR-147b	15	+	q	-2.1	2.96E-03 ↑	2		x	x	
hsa-miR-1290†	1	-	p	-2.2	3.45E-03 ↑	2		x		
hsa-miR-299-5p†	14	+	q	2.5	3.62E-03 ↓	4			x	
hsa-miR-21-5p	17	+	q	-2.2	3.66E-03 ↑	0		x		
hsa-miR-770-5p†	14	+	q	3.0	3.71E-03 ↓	3			x	
hsa-miR-1247-5p	14	-	q	2.4	4.40E-03 ↓	2		x	x	
hsa-miR-211-5p†	15	-	q	3.7	4.90E-03 ↓	4		x	x	
hsa-miR-200c-5p†	12	+	p	-2.3	5.31E-03 ↑	3		x		
hsa-miR-99a-3p	21	+	q	2.8	5.83E-03 ↓	0		x	x	
hsa-miR-486-5p	8	-	p	2.0	1.78E-02 ↓	0		x		
hsa-miR-383-5p†	8	-	p	2.7	3.05E-02 ↓	4		x		

Table S5.1. Significantly deregulated miRNAs detected using the median-imputed data. The listed miRNAs were detected as significantly deregulated (5% significance level) by BH, TST-GBH (TST), LSL-GBH (LSL), or SABHA under Scheme (c). If a method detected a miRNA, the corresponding row is marked by x. The "Chr." column gives the chromosome, the "Str." column indicates the chromosome strand, and the "arm" column specifies the arm. The "ΔΔCt" column provides the average ΔΔCt value for each miRNA across 18 patients. The "p-value" column shows raw p-values from paired t-tests. Rows are sorted by p-values, and the arrows following them denote whether the expression was upregulated (↑) or downregulated (↓) according to the sign of ΔΔCt. The "Missing pairs" column provides the number of missing pairs of observations for each miRNA.

† These miRNAs were not detected in the non-imputed data.

Table S5.2. Increase in detections after median imputation for different FDR control methods. This table reports the difference in the number of significantly deregulated miRNAs after and before imputation. For simplicity, we display results only for Schemes (a), (b), (c), and finer grouping schemes with $k = 25, 15$, and 5.

Scheme	BH	TST	LSL	SABHA
(a)	10	10	10	9
(a)	10	7	9	9
(c)	10	11	10	8
$k = 25$	10	12	13	8
$k = 15$	10	11	8	8
$k = 5$	10	12	9	9

Collectively, the group-adaptive BH methods detected 13 more miRNAs using the imputed data under our primary grouping scheme, Scheme (c) (see Table S5.1). These additional miRNAs have substantially higher missingness: on average, they have 4.62 missing tumor–control pairs, compared to the remaining miRNAs, which, except for miR-1293, have either no missingness or at most two missing pairs (Table 1). The first quartile, median, and third quartile of the number of missing pairs among these additional miRNAs are 3, 4, and 6, respectively.

Moreover, none of these additional miRNAs exhibited target genes that are significantly deregulated in the opposite direction in the whole-transcriptome analysis of Singh et al. (2017). Taken together, these observations suggest that some of these additional detections may be attributable to imputation.

Some of the additional detections after imputation had no missing tumor–control pairs (Table 1). Although this may seem surprising at first glance, it is not unexpected. As discussed in Section 2.1.1, these additional detections are likely driven by relaxed rejection thresholds resulting from imputation-induced p-value deflation. Consequently, miRNAs without missingness that did not meet the significance threshold in the original analysis may exceed the threshold after median imputation.

S6 DETAILS ON THE NOVEL MIRNAS

miR-1 (downregulated) miR-1 was downregulated in De Sarkar et al. (2014)'s data, which is consistent with its established roles as a tumor suppressor (Safa et al., 2020). The reduced expression of miR-1 in OSCC has been associated with advanced disease and poorer prognosis (Peng et al., 2017). In OSCC, miR-1 suppresses epithelial–mesenchymal transition (EMT) by directly targeting the transcription factor SNAI2 or Slug (Peng et al., 2017). SNAI2's expression was significantly upregulated with an average fold change of 2.2 in Singh et al. (2017)'s analysis. Nohata et al. (2011) reported that miR-1 targets the oncogene PNP in maxillary sinus squamous cell carcinoma (MSSCC), a subtype of SCC. Its upregulation with an average fold change of 1.7 in Singh et al. (2017) suggests the possibility that the PNP–miR-1 pathway may play a similar role in OSCC. Additional target genes of miR-1 were significantly upregulated in expression in Singh et al. (2017)'s analysis, including well-established oncogenes such as PDE7A in endometrial cancer (Yamamoto et al., 2015), CXCR4 in small cell lung cancer (Khan et al., 2023) and thyroid cancer (Leone et al., 2011), and ZNF281 in soft tissue sarcomas (Cormier and Pollock, 2004) (Table 4).

miR-21-5p (upregulated) miR-21-5p is a ubiquitous oncogene, widely proposed as a prognostic biomarker of OSCC, and the most frequently overexpressed miRNA in many cancers (Schneider et al., 2018; Dioguardi et al., 2022; Troiano et al., 2018). Therefore, its overexpression in our analysis is consistent with its well-documented oncogenic role in OSCC (Feng and Tsao, 2016; Reis et al., 2010; Reddy et al., 2024; Schneider et al., 2018).

Some earlier studies investigating the role of miR-21 in cancer referred to this miRNA without explicit 5p/3p strand specification. As discussed in Kuo et al. (2015), such strand ambiguity was common in earlier miRNA studies due to incomplete arm-feature annotation and analysis pipelines. miR-21-5p and miR-21-3p are the two mature miRNAs derived from the same precursor, miR-21; however, miR-21-5p is generally the more abundant and functionally dominant strand in cancer contexts, as documented in miRBase and large-scale sequencing studies (Kozomara et al., 2019).

PDCD4, a target gene of miR-21-5p, is associated with progression and metastasis in OSCC (cf. Reis et al., 2010; Arslan Bozdag et al., 2024), was significantly downregulated with an average fold change of 2.9 in Singh et al. (2017). PDCD4 is regarded as one of the principal targets of miR-21 (Jenike and Halushka, 2021). Table 4 shows that seven additional target genes of miR-21, namely MEF2C, TIMP3, PPARA, RECK, SPRY1, SPRY2, and THRB, were also significantly downregulated in Singh et al. (2017). According to Buscaglia and Li (2011), miR-21 suppresses the activities of these genes, whose downregulation is linked to multiple hallmarks of cancer (Hanahan and Weinberg, 2011).

miR-486-3p/5p (downregulated) miR-486-3p exhibits tumor suppressing activities in many cancers including OSCC, where its downregulation is associated with migration and invasion (ElKhouly et al., 2020; Yang et al., 2020). miR-486-5p's role in OSCC is more controversial, but recent studies support its tumor suppressing activity in OSCC, which aligns with the observed downregulation (Yan et al., 2016; Soga et al., 2013). Among the known target genes of miR-486, FLNA, MARCH1, KIAA1199, and NRP2 were significantly upregulated in Singh et al. (2017). FLNA and MARCH1 have been validated as miR-486-3p targets in laryngeal squamous cell carcinoma (ElKhouly et al., 2020) and osteosarcoma (Yang et al., 2023), respectively, whereas KIAA1199 and NRP2 were confirmed as direct miR-486-5p targets in papillary thyroid cancer (Jiao et al., 2019) and colorectal cancer (Liu et al., 2019), respectively. Among these, MARCH1 and NRP2 have been linked to prognosis in OSCC. MARCH1 modulates cell proliferation and apoptosis (Liu et al., 2022a), while NRP2 influences proliferation, migration, and invasion in OSCC (Kang et al., 2021). However, known target genes of miR-486-3p in OSCC, e.g. DDR1, ANK1, FGF44, etc., (cf. ElKhouly et al., 2020, for details) were absent from Singh et al. (2017)'s list of significantly deregulated genes.

miR-99a-3p (downregulated) miR-99a-3p has been reported to function as a tumor suppressor in several cancers, including OSCC (Chen et al., 2018; Wang et al., 2025). To our knowledge, literature on validated targets of this miRNA in OSCC is limited. However, CDK6, a validated target of miR-99b-3p in OSCC (cf. Wang et al., 2025), was found to be upregulated in Singh et al. (2017) with an average fold change of 1.7. The cancer biomarker NCAPG, identified as a direct target of miR-99a-3p in prostate cancer (Arai et al., 2018), has been shown to regulate cell proliferation, cell cycle progression, and apoptosis in OSCC (Jianing et al., 2021). In Singh et al. (2017), NCAPG was also upregulated with an average fold change of 1.7, raising the possibility that it may also be a target of miR-99a-3p in OSCC. Okada et al. (2019); Wei et al. (2019a) examined the tumor suppressor roles of miR-99a-3p in head and neck squamous cell carcinoma and identified some of its targets. Among target genes, BCAT1, MTHFD2, and RAC2 were significantly upregulated in Singh et al. (2017)'s analysis. In addition, RRM2, a direct target of miR-99a-3p in renal carcinoma (cf. Osako et al., 2019), was also found to be upregulated with average fold change of 2.7 in that study.

miR-1247-5p (downregulated) miR-1247-5p typically acts as a tumor suppressor in several cancers, which is consistent with our observations (Chu et al., 2017; Yi et al., 2017; Zhang et al., 2016; Wei et al., 2019b). Although research on the role of miR-1247 in OSCC is limited (Liu et al., 2020), it has been found to be downregulated in cutaneous squamous cell carcinoma, a subtype of SCC (An et al., 2019). Moreover, the oncogene STMN1, reported as a functional target of miR-1247 in non-small-cell lung cancer (Zhang et al., 2016), was significantly upregulated (average fold change 1.5) in the analysis of Singh et al. (2017).

miR-455-3p (upregulated) MiR-455-3p has been studied in several cancers, and its role is context-dependent. Broadly, it has been reported to act as both a tumor suppressor and an oncogene, depending on the tissue context (Ye et al., 2023). Some recent studies have highlighted its oncogenic roles in OSCC, in line with our observations (Li et al., 2020; Liu et al., 2022b). Liu et al. (2022b) showed that miR-455-3p targets the tumor suppressor transcription factor ELF3 in OSCC, which was significantly downregulated (average fold change 4.8) in Singh et al. (2017)'s analysis. miR-455-3p's oncogenic activity and elevated expression have also been reported in other carcinomas, including esophageal squamous cell carcinoma (Liu et al., 2017), colorectal cancer (Ye et al., 2023), and triple-negative breast cancer (Li et al., 2016).

miR-455-5p (upregulated) miR-455-5p is suspected to promote cancer cell migration and invasion in OSCC, which aligns with the observed upregulation (Hsiao et al., 2023; Cheng et al., 2016). Wu et al. (2021) suggested that miR-455-5p modulates cell proliferation and growth in OSCC by targeting PTPRS. PTPRS was significantly downregulated with an average fold change of 2.9 in Singh et al. (2017). This miRNA also exhibits oncogenic activities in breast cancer (Aili et al., 2018), lung cancer (Wang et al., 2017), as well as bladder urothelial carcinoma (Hamilton et al., 2013). Aili et al. (2018) showed that miR-455-5p targets the tumor suppressor gene PDCD4 in breast cancer, which was significantly downregulated with an average fold change of 2.9 in Singh et al. (2017). PDCD4, also a primary target of miR-21-5p, is associated with nodal metastasis and invasion in OSCC (Reis et al., 2010).

REFERENCES

Aili, T., Paizula, X., and Ayoufu, A. (2018). miR-455-5p promotes cell invasion and migration in breast cancer. *Molecular Medicine Reports*, 17(1):1825–1832.

An, X., Liu, X., Ma, G., and Li, C. (2019). Upregulated circular RNAcirc_0070934 facilitates cutaneous squamous cell carcinoma cell growth and invasion by sponging miR-1238 and miR-1247–5p. *Biochemical and Biophysical Research Communications*, 513(2):380–385.

Arai, T., Okato, A., Yamada, Y., Sugawara, S., Kurozumi, A., Kojima, S., Yamazaki, K., Naya, Y., Ichikawa, T., and Seki, N. (2018). Regulation of NCAPG by miR-99a-3p (passenger strand) inhibits cancer cell aggressiveness and is involved in CRPC. *Cancer medicine*, 7(5):1988–2002.

Arslan Bozdag, L., Açik, L., Ersoy, H. E., Bayir, Ö., Korkmaz, M. H., Mollaoglu, N., and Gultekin, S. E. (2024). Pdcd 4 and mir-21 are promising biomarkers in the follow-up of oed in liquid biopsies. *Oral Diseases*, 30(6):3873–3883.

Buscaglia, L. E. B. and Li, Y. (2011). Apoptosis and the target genes of microrna-21. *Chinese journal of cancer*, 30(6):371.

Chen, L., Hu, J., Pan, L., Yin, X., Wang, Q., and Chen, H. (2018). Diagnostic and prognostic value of serum miR-99a expression in oral squamous cell carcinoma. *Cancer Biomarkers*, 23(3):333–339.

Cheng, C.-M., Shiah, S.-G., Huang, C.-C., Hsiao, J.-R., and Chang, J.-Y. (2016). Up-regulation of miR-455-5p by the TGF-β–SMAD signalling axis promotes the proliferation of oral squamous cancer cells by targeting ube2b. *The Journal of pathology*, 240(1):38–49.

Chu, Y., Fan, W., Guo, W., Zhang, Y., Wang, L., Guo, L., Duan, X., Wei, J., and Xu, G. (2017). miR-1247-5p functions as a tumor suppressor in human hepatocellular carcinoma by targeting wnt3. *Oncology reports*, 38(1):343–351.

Cormier, J. N. and Pollock, R. E. (2004). Soft tissue sarcomas. *CA: a cancer journal for clinicians*, 54(2):94–109.

De Sarkar, N., Roy, R., Mitra, J. K., Ghose, S., Chakraborty, A., Paul, R. R., Mukhopadhyay, I., and Roy, B. (2014). A quest for miRNA bio-marker: a track back approach from gingivo buccal cancer to two different types of precancers. *PLOS One*, 9(8):e104839.

Dioguardi, M., Spirito, F., Sovereto, D., Alovisi, M., Troiano, G., Aiuto, R., Garcovich, D., Crincoli, V., Laino, L., Cazzolla, A. P., and Lo Muzio, L. (2022). MicroRNA-21 expression as

a prognostic biomarker in oral cancer: Systematic review and meta-analysis. *International Journal of Environmental Research and Public Health*, 19(6):3396.

ElKhouly, A. M., Youness, R., and Gad, M. (2020). Microrna-486-5p and microrna-486-3p: Multifaceted pleiotropic mediators in oncological and non-oncological conditions. *Non-coding RNA research*, 5(1):11–21.

Feng, Y.-H. and Tsao, C.-J. (2016). Emerging role of microrna-21 in cancer. *Biomedical reports*, 5(4):395–402.

Hamilton, M. P., Rajapakshe, K., Hartig, S. M., Reva, B., McLellan, M. D., Kandoth, C., Ding, L., Zack, T. I., Gunaratne, P. H., Wheeler, D. A., and The Cancer Genome Atlas Research Network (2013). Identification of a pan-cancer oncogenic microRNA superfamily anchored by a central core seed motif. *Nature Communications*, 4(1):2730.

Hanahan, D. and Weinberg, R. A. (2011). Hallmarks of cancer: the next generation. *cell*, 144(5):646–674.

Hsiao, S.-Y., Weng, S.-M., Hsiao, J.-R., Wu, Y.-Y., Wu, J.-E., Tung, C.-H., Shen, W.-L., Sun, S.-F., Huang, W.-T., Lin, C.-Y., and Chang, J.-Y. (2023). MiR-455-5p suppresses PDZK1IP1 to promote the motility of oral squamous cell carcinoma and accelerate clinical cancer invasion by regulating partial epithelial-to-mesenchymal transition. *Journal of Experimental & Clinical Cancer Research*, 42(1):40.

Jenike, A. E. and Halushka, M. K. (2021). mir-21: a non-specific biomarker of all maladies. *Biomarker Research*, 9(1):1–7.

Jianing, L., Shiqun, S., Jia, L., Xuetao, Z., Zehua, L., Tong, S., and Zhi, C. (2021). Ncapg, mediated by miR-378a-3p, regulates cell proliferation, cell cycle progression, and apoptosis of oral squamous cell carcinoma through the GSK-3β/β-catenin signaling. *Neoplasma*, 68(6).

Jiao, X., Ye, J., Wang, X., Yin, X., Zhang, G., and Cheng, X. (2019). Kiaa1199, a target of micorna-486-5p, promotes papillary thyroid cancer invasion by influencing epithelial-mesenchymal transition (emt). *Medical Science Monitor: International Medical Journal of Experimental and Clinical Research*, 25:6788.

Kang, Y., Zhang, Y., Zhang, Y., and Sun, Y. (2021). Nrp2, a potential biomarker for oral squamous cell carcinoma. *American Journal of Translational Research*, 13(8):8938.

Khan, P., Siddiqui, J. A., Kshirsagar, P. G., Venkata, R. C., Maurya, S. K., Mirzapoiazova, T., Perumal, N., Chaudhary, S., Kanchan, R. K., Fatima, M., Mallya, K., Atri, P., Sharma, S., Shcherbinin, D., Seshacharyulu, P., Jain, M., Ponnusamy, M. P., Natarajan, A., Joshi, S. S., and Batra, S. K. (2023). MicroRNA-1 attenuates the growth and metastasis of small cell lung cancer through CXCR4/FOXM1/RRM2 axis. *Molecular Cancer*, 22(1):1.

Kozomara, A., Birgaoanu, M., and Griffiths-Jones, S. (2019). miRBase: from microrna sequences to function. *Nucleic acids research*, 47(D1):D155–D162.

Kuo, W.-T., Su, M.-W., Lee, Y. L., Chen, C.-H., Wu, C.-W., Fang, W.-L., Huang, K.-H., and Lin, W.-c. (2015). Bioinformatic interrogation of 5p-arm and 3p-arm specific miRNA expression using tcga datasets. *Journal of Clinical Medicine*, 4(9):1798–1814.

Leone, V., D'Angelo, D., Rubio, I., de Freitas, P. M., Federico, A., Colamaio, M., Pallante, P., Medeiros-Neto, G., and Fusco, A. (2011). MiR-1 Is a Tumor Suppressor in Thyroid Carcinogenesis Targeting CCND2, CXCR4, and SDF-1alpha. *The Journal of Clinical Endocrinology & Metabolism*, 96(9):E1388–E1398.

Li, Q., Sun, Q., and Zhu, B. (2020). Lncrna xist inhibits the progression of oral squamous cell carcinoma via sponging mir-455-3p/BTG2 axis. *OncoTargets and therapy*, pages 11211–11220.

Li, Z., Meng, Q., Pan, A., Wu, X., Cui, J., Wang, Y., and Li, L. (2016). Microrna-455-3p promotes invasion and migration in triple negative breast cancer by targeting tumor suppressor EI24. *Oncotarget*, 8(12):19455.

Liu, A., Liu, L., and Lu, H. (2019). Lncrna xist facilitates proliferation and epithelial–mesenchymal transition of colorectal cancer cells through targeting mir-486-5p and promoting neuropilin-2. *Journal of cellular physiology*, 234(8):13747–13761.

Liu, A., Zhu, J., Wu, G., Cao, L., Tan, Z., Zhang, S., Jiang, L., Wu, J., Meng, L., Song, L., and Li, J. (2017). Antagonizing mir-455-3p inhibits chemoresistance and aggressiveness in esophageal squamous cell carcinoma. *Molecular Cancer*, 16(1):106.

Liu, K. Y. P., Zhu, S. Y., Brooks, D., Bowlby, R., Durham, J. S., Ma, Y., Moore, R. A., Mungall, A. J., Jones, S., and Poh, C. F. (2020). Tumor microrna profile and prognostic value for lymph node metastasis in oral squamous cell carcinoma patients. *Oncotarget*, 11(23):2204.

Liu, L., Guo, B., Han, Y., Xu, S., and Liu, S. (2022a). March1 silencing suppresses growth of oral squamous cell carcinoma through regulation of PHLPP2. *Clinical and Translational Oncology*, 24(7):1311–1321.

Liu, X., Ma, X., Li, H., Wang, Y., Mao, M., Liang, C., and Hu, Y. (2022b). Linc00472 suppresses oral squamous cell carcinoma growth by targeting miR-455-3p/ELF3 axis. *Bioengineered*, 13(1):1162–1173.

Nohata, N., Hanazawa, T., Kikkawa, N., Sakurai, D., Sasaki, K., Chiyomaru, T., Kawakami, K., Yoshino, H., Enokida, H., Nakagawa, M., and Seki, N. (2011). Identification of novel molecular targets regulated by tumor suppressive miR-1/miR-133a in maxillary sinus squamous cell carcinoma. *International Journal of Oncology*, 39(5):1099–1107.

Okada, R., Koshizuka, K., Yamada, Y., Moriya, S., Kikkawa, N., Kinoshita, T., Hanazawa, T., and Seki, N. (2019). Regulation of oncogenic targets by miR-99a-3p (passenger strand of mir-99a-duplex) in head and neck squamous cell carcinoma. *Cells*, 8(12):1535.

Osako, Y., Yoshino, H., Sakaguchi, T., Sugita, S., Yonemori, M., Nakagawa, M., and Enokida, H. (2019). Potential tumor-suppressive role of microrna-99a-3p in sunitinib-resistant renal cell carcinoma cells through the regulation of rrm2. *International journal of oncology*, 54(5):1759–1770.

Peng, C.-Y., Liao, Y.-W., Lu, M.-Y., Yu, C.-H., Yu, C.-C., and Chou, M.-Y. (2017). Downregulation of miR-1 enhances tumorigenicity and invasiveness in oral squamous cell carcinomas. *Journal of the Formosan Medical Association*, 116(10):782–789.

Reddy, C. S. S., PP, A. S. U., Ganapathy, D. M., KP, A., and Sekar, D. (2024). Microrna-21 as a biomarker in terminal stage oral squamous cell carcinoma (oscc) in the south indian population. *Oral Oncology Reports*, 9:100139.

Reis, P. P., Tomenson, M., Cervigne, N. K., Machado, J., Jurisica, I., Pintilie, M., Sukhai, M. A., Perez-Ordonez, B., Grénman, R., Gilbert, R. W., Gullane, P. J., Irish, J. C., and Kamel-Reid, S. (2010). Programmed cell death 4 loss increases tumor cell invasion and is regulated by miR-21 in oral squamous cell carcinoma. *Molecular Cancer*, 9(1):238.

Safa, A., Bahroudi, Z., Shoorei, H., Majidpoor, J., Abak, A., Taheri, M., and Ghafouri-Fard, S. (2020). mir-1: A comprehensive review of its role in normal development and diverse disorders. *Biomedicine & Pharmacotherapy*, 132:110903.

Schneider, A., Victoria, B., Lopez, Y. N., Suchorska, W., Barczak, W., Sobecka, A., Golusinski, W., Masternak, M. M., and Golusinski, P. (2018). Tissue and serum microrna profile of oral squamous cell carcinoma patients. *Scientific reports*, 8(1):675.

Singh, R., De Sarkar, N., Sarkar, S., Roy, R., Chattopadhyay, E., Ray, A., Biswas, N. K., Maitra, A., and Roy, B. (2017). Analysis of the whole transcriptome from gingivo-buccal squamous cell carcinoma reveals deregulated immune landscape and suggests targets for immunotherapy. *PLOS One*, 12(9):e0183606.

Soga, D., Yoshiba, S., Shiogama, S., Miyazaki, H., Kondo, S., and Shintani, S. (2013). microrna expression profiles in oral squamous cell carcinoma. *Oncology reports*, 30(2):579–583.

Troiano, G., Mastrangelo, F., Caponio, V., Laino, L., Cirillo, N., and Lo Muzio, L. (2018). Predictive prognostic value of tissue-based microrna expression in oral squamous cell carcinoma: a systematic review and meta-analysis. *Journal of dental research*, 97(7):759–766.

Wang, B., Howel, P., Bruheim, S., Ju, J., Owen, L. B., Fodstad, O., and Xi, Y. (2011). Systematic evaluation of three microrna profiling platforms: microarray, beads array, and quantitative real-time pcr array. *PLOS One*, 6(2):e17167.

Wang, J., Wang, Y., Sun, D., Bu, J., Ren, F., Liu, B., Zhang, S., Xu, Z., Pang, S., and Xu, S.

(2017). miR-455-5p promotes cell growth and invasion by targeting SOCO3 in non-small cell lung cancer. *Oncotarget*, 8(70):114956.

Wang, Y., Huang, D., Li, M., and Yang, M. (2025). Microrna-99 family in cancer: molecular mechanisms for clinical applications. *PeerJ*, 13:e19188.

Wei, G.-G., Guo, W.-P., Tang, Z.-Y., Li, S.-H., Wu, H.-Y., and Zhang, L.-C. (2019a). Expression level and prospective mechanism of miRNA-99a-3p in head and neck squamous cell carcinoma based on miRNA-chip and miRNA-sequencing data in 1, 167 cases. *Pathology-Research and Practice*, 215(5):963–976.

Wei, Q., Yao, J., and Yang, Y. (2019b). Microrna-1247 inhibits the viability and metastasis of osteosarcoma cells via targeting nrp1 and mediating wnt/β-catenin pathway. *Eur Rev Med Pharmacol Sci*, 23(17):7266–7274.

Wu, J., Li, Y., Liu, J., and Xu, Y. (2021). Down-regulation of lncrna hcg11 promotes cell proliferation of oral squamous cell carcinoma through sponging miR-455-5p. *The Journal of Gene Medicine*, 23(3):e3293.

Yamamoto, N., Nishikawa, R., Chiyomaru, T., Goto, Y., Fukumoto, I., Usui, H., Mitsuhashi, A., Enokida, H., Nakagawa, M., Shozu, M., and Seki, N. (2015). The tumor-suppressive microRNA-1/133a cluster targets PDE7A and inhibits cancer cell migration and invasion in endometrial cancer. *International Journal of Oncology*, 47(1):325–334.

Yan, Y., Wang, X., Venø, M. T., Bakholdt, V., Sørensen, J. A., Krogdahl, A., Sun, Z., Gao, S., and Kjems, J. (2016). Circulating miRNAs as biomarkers for oral squamous cell carcinoma recurrence in operated patients. *Oncotarget*, 8(5):8206.

Yang, H., He, C., Feng, Y., and Jin, J. (2023). Exosome-delivered mir-486-3p inhibits the progression of osteosarcoma via sponging CircKEAP1/MARCH1 axis components. *Oncology Letters*, 27(1):24.

Yang, H., Huang, Y., He, J., Chai, G., Di, Y., Wang, A., and Gui, D. (2020). Mir-486-3p inhibits the proliferation, migration and invasion of retinoblastoma cells by targeting ecm1. *Bioscience Reports*, 40(6).

Ye, L., Fan, T., Qin, Y., Qiu, C., Li, L., Dai, M., Zhou, Y., Chen, Y., and Jiang, Y. (2023). Microrna-455-3p accelerate malignant progression of tumor by targeting H2AFZ in colorectal cancer. *Cell Cycle*, 22(7):777–795.

Yi, J. M., Kang, E.-J., Kwon, H.-M., Bae, J.-H., Kang, K., Ahuja, N., and Yang, K. (2017). Epigenetically altered miR-1247 functions as a tumor suppressor in pancreatic cancer. *Oncotarget*, 8(16):26600.

Zhang, J., Fu, J., Pan, Y., Zhang, X., and Shen, L. (2016). Silencing of miR-1247 by DNA methylation promoted non-small-cell lung cancer cell invasion and migration by effects of stmn1. *OncoTargets and therapy*, pages 7297–7307.
